# *Alu *pair exclusions in the human genome

**DOI:** 10.1186/1759-8753-2-10

**Published:** 2011-09-23

**Authors:** George W Cook, Miriam K Konkel, James D Major, Jerilyn A Walker, Kyudong Han, Mark A Batzer

**Affiliations:** 1 Department of Biological Sciences, Louisiana State University, Baton Rouge, Louisiana, 70803, USA; 2 Department of Nanobiomedical Science & WCU Research Center, Dankook University, Chungnam 330-714, Republic of Korea

## Abstract

**Background:**

The human genome contains approximately one million *Alu *elements which comprise more than 10% of human DNA by mass. *Alu *elements possess direction, and are distributed almost equally in positive and negative strand orientations throughout the genome. Previously, it has been shown that closely spaced *Alu *pairs in opposing orientation (inverted pairs) are found less frequently than *Alu *pairs having the same orientation (direct pairs). However, this imbalance has only been investigated for *Alu *pairs separated by 650 or fewer base pairs (bp) in a study conducted prior to the completion of the draft human genome sequence.

**Results:**

We performed a comprehensive analysis of all (> 800,000) full-length *Alu *elements in the human genome. This large sample size permits detection of small differences in the ratio between inverted and direct *Alu *pairs (I:D). We have discovered a significant depression in the full-length *Alu *pair I:D ratio that extends to repeat pairs separated by ≤ 350,000 bp. Within this imbalance bubble (those *Alu *pairs separated by ≤ 350,000 bp), direct pairs outnumber inverted pairs. Using PCR, we experimentally verified several examples of inverted *Alu *pair exclusions that were caused by deletions.

**Conclusions:**

Over 50 million full-length *Alu *pairs reside within the I:D imbalance bubble. Their collective impact may represent one source of *Alu *element-related human genomic instability that has not been previously characterized.

## Background

Retrotransposons are mobile DNA elements that populate genomes via their respective RNA transcripts. The retrotransposon with the highest copy number in the human genome is the *Alu *element [1]. *Alu *elements lack the necessary repertoire of enzymes to effect their independent insertion and are thus classified as non-autonomous mobile elements. For recent reviews, see [2-4].

Following transcription, *Alu *RNA is thought to require the assistance of the LINE1 open reading frame 2 protein (ORF2p) both for nicking the genome at the insertion site and for reverse transcription of the *Alu *RNA transcript [5,6]. The endonuclease and reverse transcriptase functions of ORF2p are referred to as L1EN and L1RT, respectively. While L1EN has been shown to have some tolerance for target site variation, it most frequently cleaves at the T/A transition within the sequence, 5'-TTTTAA-3' [7-10]. Following cleavage, the poly-T sequence of the target site becomes accessible to the complementary poly(A) tail of *Alu *RNA. Hybridization of these two sequences results in a short RNA-DNA hybrid that both orients the RNA transcript and primes reverse transcription of the *Alu *RNA by L1RT. Identical sequences flanking the insertion are characteristic of most *Alu *elements [11]. These flanking sequences are referred to as target site duplications (TSDs) [2,12]. The presence of TSDs suggests that a nick occurs on the complementary strand of DNA 3' to the L1EN cleavage site on the first strand. However, little is known of the mechanisms associated with this second nick or the eventual insertion of the 5' end of the *Alu *element [13,14]. This process of *Alu *element mobilization and insertion is commonly referred to as target primed reverse transcription (TPRT) [15,16]. TPRT also occurs with two additional non-long terminal repeat (LTR) retrotransposons, LINE1 and SVA (SINE-R, variable number of tandem repeats and *Alu*) elements, within the human lineage [8]. While recognizing rare exceptions [17,18], the majority of non-LTR retrotransposon insertions are dependent upon the activity of L1EN. As with *Alu *elements, LINE1 and SVA element insertions are typically characterized by TSDs that flank each element.

*Alu *elements also possess several features that provide directionality. Including the poly(A) tail, full-length *Alu *elements are approximately 300 bp in length (Additional File 1, Figure S1) and are dimeric structures with two adenine-rich regions flanking the 3' monomer [2,19]. The middle adenine-rich region separates the two monomers and the 3' adenine-rich region forms the variable length poly(A) tail. Additionally, the 5' monomer possesses the A and B boxes required for the transcription by RNA Polymerase III and the 3' monomer contains a 31-bp insert not present in the 5' monomer [20,21].

Inverted pairs of full-length *Alu *elements form near-palindromic sequences that are separated by spacers of other DNA sequences of varying size and composition. Palindromic sequences have been shown to be unstable in *Escherichia coli *[22], yeast [23] and mice [24]. The genomic instability of inverted *Alu *pairs has also been demonstrated in a yeast experimental system [25]. Other previous research has reported that inverted *Alu *pairs are potential sources of chromosomal instability when separated by ≤ 650 bp in humans [26]. The ability of *Alu *sequences to interact is directly correlated with the degree of sequence identity between the copies [25]. It is estimated that the majority of full-length human *Alu *elements share sequence identity ranging between 65 and 85 percent [26].

*Alu *element insertions have been linked to several genetic diseases including hemophilia, hypercholesterolemia and various cancers [4,27]. While multiple diseases have been attributed to *Alu *element insertions, their most important role may be in shaping human genome architecture through various post-insertion interactions. Such interactions could result in deletions, duplications, inversions and a host of other complex genomic structural changes [9,28]. *Alu *element interactions with each other have been found to generate recombination mediated deletions and inversions [10,29,30]. In addition, *Alu *elements have been associated with multiple deletions related to various cancers [8,31] and copy number variation breakpoints [9,32-35].

It has also been shown in humans that closely spaced adjacent *Alu *pairs in opposing orientation (inverted pairs) are found less frequently than *Alu *pairs having the same orientation (direct pairs) [26]. However, this imbalance has previously only been investigated for *Alu *pairs separated by ≤ 650 bp in a study conducted prior to the completion of the draft human genome sequence. Here, we have performed a comprehensive analysis of all (> 800,000) full-length *Alu *elements (275 to 325 bp) in the public human genome assembly (hg18). Using the large data set of full-length *Alu *elements enabled us to detect small imbalances in the ratio between inverted and direct *Alu *pairs (I:D). We report a potential new insight into human genomic instability, a non-random depression in the I:D ratio for full-length *Alu *pairs whose elements are separated by up to 350,000 bp (*P *< 0.05). Over 50 million (59,357,435) full-length *Alu *pairs reside within this I:D imbalance window. This phenomenon of full-length *Alu *pair I:D imbalance is hypothesized to reflect the activity of four separate mechanisms which result in *Alu *pair exclusions (APEs).

## Results

The size distribution of the human genomic *Alu *element population is shown in Additional File 1, Figure S1. Full-length *Alu *elements, having lengths between 275 and 325 bp, account for approximately 69 percent of all human *Alu *elements. Slightly over two percent of human *Alu *elements have lengths greater than 325 bp with 29 percent being truncated (< 275 bp). Sequences of less than 30 bp cannot be reliably determined to be actual *Alu *elements and are therefore excluded from this study (*P *< 0.05). *Alu *element length constraints provide a full-length *Alu *element sample size of 806,880 (Methods).

The directionality of *Alu *elements creates four possible types of *Alu *pairs (Additional File 1, Figure S2). Two of these four configurations share both elements in the same (or direct) orientation and two share elements in the opposite (or inverted) orientation. A pair of *Alu *elements in which both members of the pair are positioned on the positive strand are in the 'forward' orientation. Conversely, when both members in the pair are positioned on the negative strand, the pair is defined as being in the 'reverse' orientation. Throughout this manuscript, the sequence separating each pair is referred to as the spacer. When an inverted *Alu *pair is oriented with the poly(A) tails pointing toward each other, the pair is termed as being in the 'tail-to-tail' orientation, and when an inverted pair is oriented with the poly(A) tails pointing away from each other, it is termed as being in the 'head-to-head' orientation.

### I:D ratio for adjacent full-length *Alu *pairs departs from unity

Departures from unity in the full-length *Alu *pair (FAP) I:D ratio may be suggestive of non-random insertion or deletion of *Alu *elements within the human genome. Testing for randomness was performed using binomial distributions assuming an equal probability for *Alu *insertions to occur on both the positive and negative strands (Methods). Adjacent FAPs contain no *Alu *elements within the spacer. The human adjacent FAP population of 560,485 contains 252,748 inverted pairs and 307,737 direct pairs. The I:D ratio for this population is 0.8213. Any I:D ratio outside of 0.9947 to 1.0053 reflects a non-random distribution (*P *< 0.05). The I:D ratio for adjacent FAPs of 0.8213 represents a *P*-value of < 0.000001 and therefore falls well outside of the 95 percent confidence interval for randomness.

Furthermore, the adjacent FAP I:D ratio departure from unity appears to be a function of the FAP spacer size. The median spacer size for adjacent FAPs is 930 bp (mean spacer length = 921 bp). Adjacent FAPs with less than and greater than this median spacer length possess I:D ratios of 0.7105 and 0.9477, respectively. The expected I:D range for a random distribution of these half-size FAP populations is 0.9925 to 1.0075 (*P *< 0.05). A more thorough analysis of the variation of FAP I:D ratio versus spacer size requires adjustment of the data set and is provided later in this section (see CLIQUES, catenated L1EN induced queues of uninterrupted *Alu*, LINE1 and SVA elements).

The adjacent FAP I:D imbalance calculation reported above provides a macroscopic view of the entire human genome. Human chromosome one was chosen to determine if a similar I:D bias (non-random distributions of *Alu *elements with respect to orientation) was evident across a smaller region of the genome. A comparison of the actual distribution versus a simulated random distribution of *Alu *elements on chromosome one indicated that orientational clustering of *Alu *elements occurs over 40 percent more frequently than would be expected if *Alu *insertions were orientationally random (Additional File 1, Figure S3).

### Three patterns of I:D ratio

**Figure 1:**
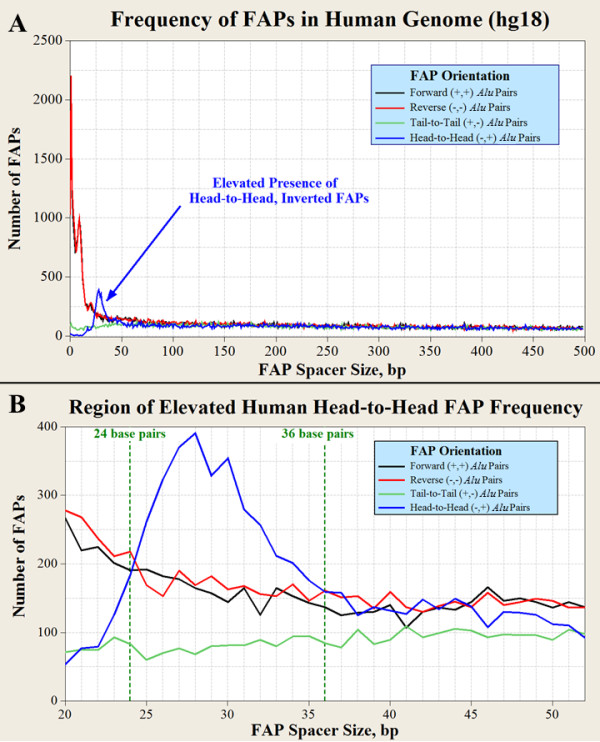
**Frequency of closely spaced FAPs**. **(A) **Human adjacent FAP frequency versus the spacer size (bp) separating the two members of the FAP. The number of inverted pairs (blue and green lines) is much lower than the number of direct pairs (red and black lines) when the spacer has a size ≤ 24 bp (I:D = 0.076); **(B) **Spacer lengths within 24 to 36 bp define the only region within the human genome where head-to-head (inverted) FAPs outnumber either type of direct oriented FAPs. Bp: base pair; FAP: full-length *Alu *pair.

Figure 1A illustrates the I:D ratio for adjacent human FAPs which are separated by ≤ 500 bp. This range includes over one-third of the human adjacent FAP population and is the first breakdown of this I:D parameter by individual spacer length. Three distinct patterns of FAP density and I:D ratio are evident from Figure 1.

The first pattern is the combined high FAP density and low I:D ratio (0.073) for spacer lengths of ≤ 24 bp. An unexpected inflection point in the frequency of direct FAPs occurs after as spacer size of 6 bp (Figure 1A). This pattern may be indicative of a potential orientational insertion preference for *Alu *elements within the TSD of an existing *Alu *element. The second FAP I:D ratio pattern evident in Figure 1A (magnified in Figure 1B) is the 13 bp span of elevated FAPs in the head-to-head orientation within the spacer size range of 24 to 36 bp. This span contains 1.6 percent of adjacent human FAPs and is the only spacer size range within the human genome where the FAP I:D ratio exceeds unity (I:D = 1.053). Previous research identified an elevated presence of *Alu *pairs (> 275 bp) in this orientation for the spacer size range of 21 to 40 bp [26]. As can be seen in Figure 1B, the most accentuated head-to-head frequencies occur between spacer lengths of 24 to 36 bp. For this span of spacer sizes, head-to-head (inverted) FAPs outnumber either forward or reverse (direct) FAPs. Although the most elevated head-to-head frequencies reside within the spacer size range of 24 to 36 bp, Figure 1B also reveals that an attenuated elevation of head-to-head FAPs over tail-to-tail inverted FAPs is present within the spacer size range of 37 to 50 bp.

The third FAP density and I:D ratio pattern is evident in Figure 1A. It is characterized by similar FAP frequencies among the four *Alu *pair types between spacer sizes of 51 to 500 bp. This third pattern persists for adjacent FAPs with spacer sizes of > 500 bp (data not shown).

### CLIQUEs, catenated L1EN induced queues of uninterrupted *Alu*, LINE1 and SVA elements

The common dependence of *Alu*, L1NE1, and SVA insertions upon L1 enzymes raises the possibility that the clustering of closely spaced *Alu *elements (≤ 50 bp) observed in Figure 1A is also associated with various combinations of all three element types. A similar clustering pattern exists in the form of catenated *Alu *clusters (see Additional File 1, Catenated *Alu *Clusters and Figure S4). A total of 412,380 various combinations of these *Alu*-LINE1-SVA clusters are present within the human genome. These clusters comprise 16.6 percent of all human DNA and contain 52.6 percent of the *Alu*, LINE1 and SVA sequence within the human genome. Retrotransposons residing within these L1EN-induced clusters can exist in both orientations but exhibit a clear bias for one orientation. These clusters are characterized by this orientational bias as the I:D ratio for adjacent FAPs within these clusters is 0.3847. These clusters are enriched with potential L1EN target sites because of their shared TPRT insertion mechanism creating L1EN-induced TSDs flanking these three types of retrotransposons, as well as by the adenine-rich region within *Alu *elements (see Discussion, APE mechanisms). This enrichment of potential L1EN target sites inherently increases the likelihood of future *Alu*, LINE1 and SVA elements within these clusters. The common participation of *Alu*, L1NE1, and SVA elements within catenated clusters is consistent with L1EN activity. These catenated L1EN induced queues of uninterrupted *Alu*, L1NE1, and SVA elements are hereafter referred to as CLIQUEs.

The potential for TPRT-related insertion bias within TSDs makes CLIQUE identification an important consideration in evaluating deviations from unity in the FAP I:D ratio. The potential for L1EN orientational bias to propagate within CLIQUEs could conceivably result in FAPs separated by more than 10 kb to be orientationally related. As an example, CLIQUE number 397,134 (chrX:74,530,726-74,548,236) is 17,511 bp in length and contains two full-length *Alu *elements which form a FAP in the forward orientation with a spacer size of 11,870 bp. This potential for orientational bias between *Alu *elements residing within the same CLIQUE has resulted in their exclusion for determination of genome-wide FAP I:D ratios. The adjacent FAP I:D ratio, excluding FAPs generated within the same CLIQUE, reduces the FAP sample size from 560,485 to 460,588. This correction increases the adjacent FAP I:D ratio from 0.821 to 0.955. The smaller sample size for CLIQUE corrected adjacent FAPs slightly decreases the precision for detection of non-random I:D ratios from 0.9947 to 1.0053 to 0.9942 to 1.0058 (*P *< 0.05). However, the CLIQUE-adjusted adjacent I:D ratio (0.955) remains statistically different from random (*P *< 0.00001) even though it varies with spacer size. The most closely spaced 10 percent of human adjacent FAPs (spacer size = 51-205 bp) have an I:D ratio of 0.898 while the most distantly spaced 10 percent (spacer size = approximately 7,400-50,000 bp) have an I:D ratio of 0.989. This relationship is illustrated in Additional File 1, Figure S6.

A calculated 52.6 percent of human LINE1, *Alu *and SVA sequences reside in CLIQUEs. The average CLIQUE is 1,169 bp in length and is occupied by 3.3 elements. The median CLIQUE length is 638 bp and 95 percent of all CLIQUEs have lengths less than 4,100 bp. The most CLIQUE-rich chromosome is the chromosome 19 (0.252 CLIQUES per kb) and the least rich is chromosome Y (0.061 CLIQUEs per kb). Over half of the longest 100 CLIQUEs are found on chromosome X, with the longest being over 55,000 bp at locus chrX:75,592,945-75,648,671 (Additional File 1, Figure S5).

### Non-adjacent *Alu *pair

**Figure 2:**
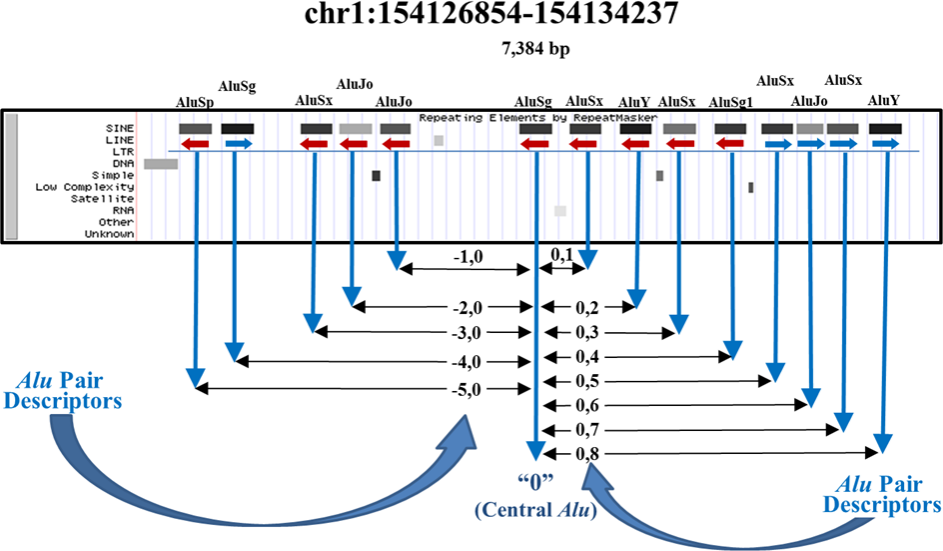
**Naming convention for FAPs**. This example from chr1:154,126,854-154,134,237 (7,384 bp) illustrates the FAP naming convention. The central *Alu *is always the element being evaluated and the second member of the pair is designated by its sequential separation from the central *Alu*. The central *Alu *is designated with the number '0'. The absolute value of the sequential separation of a given *Alu *element from the central *Alu *is defined as its APSN. Additionally, Alu elements located 5' of the central *Alu *are assigned a negative value and with a positive value if located 3' of the central *Alu*. APSN: *Alu *pair sequence number; FAP: full-length *Alu *pair.

One of the findings in this study is that the FAP I:D imbalance is not limited to adjacent FAPs. Intervening *Alu *elements within the spacer of a FAP also generate non-random FAP I:D ratios. This non-random I:D imbalance (*P *< 0.05) was detected in FAPs whose spacer contains up to 106 intervening *Alu *elements and over 350,000 bp. Taken at the whole human genome level, the human FAP I:D imbalance window encompasses ± 107 of an *Alu*'s neighboring *Alu *elements (Methods). No size constraint was placed upon intervening *Alu *elements. Therefore, while the entire inventory of human *Alu *elements is used in this study, only I:D ratios for FAPs are reported. The smallest CLIQUE adjusted FAP sample size (460,588) occurs for adjacent FAPs. Sample size ranges of 551,764 to 557,454 exist for all FAP families with more than three intervening *Alu *elements within the spacer (Additional File 1, Table S1). The inclusion of FAPs with intervening *Alu *elements requires terminology for defining different FAP types (Figure 2 and Methods).

### I:D ratio versus *Alu *pair sequence number

Adjusting the adjacent (0,1) FAP population for CLIQUEs increases its median spacer size from 930 to 1,296 bp. The CLIQUE-adjusted I:D ratios for the smaller and larger spacer sizes about this new median are 0.951 and 0.959, respectively. Both of these I:D ratios are outside of the 0.9918 to 1.0082 range which would be expected for a random distribution (*P *< 0.05). The small difference between these I:D ratios raises the possibility that FAPs with much larger spacers may also be subject to an FAP I:D imbalance. Unfortunately, this hypothesis is difficult to measure using only adjacent FAPs as 95 percent of this population has spacer sizes of less than 11,005 bp.

**Figure 3:**
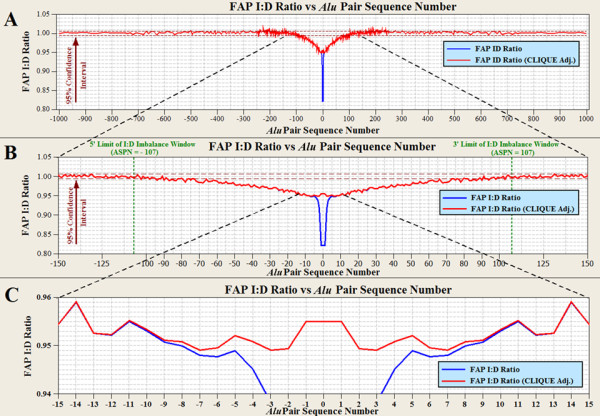
**FAP I:D ratio versus *Alu *pair sequence number**. The APSN, with (red) and without (blue) correction for CLIQUEs. **(A) **The I:D ratio of full length *Alu *pairs for APSNs of ± 1,000 *Alu *elements. Note that a bubble of depressed I:D ratio exists for those elements within about ± 100 *Alu *elements of the central *Alu *element. **(B) **A closer view of the I:D imbalance bubble. The 95% confidence for each value is estimated ± 0.6%. Therefore, the bubble of I:D imbalance extends for an approximately APSN = ± 85 around the central *Alu*. A more rigorous treatment of the data (see text) extends this I:D imbalance boundary to an APSN = ± 107. **(C) **Over 99% of the impact of CLIQUEs on the FAP I:D ratio dissipates after the APSN = 5. The largest CLIQUEs, while rare, contain up to 32 *Alu *elements. No CLIQUE impact exists on the FAP I:D ratio for an APSN > 31. APSN: *Alu *pair sequence number; CLIQUE: catenated LINE1 endonuclease induced queue of uninterrupted *Alu*, LINE1 and SVA elements; FAP: full-length *Alu *pair; I:D Ratio: ratio between inverted and direct *Alu *pairs.

The inclusion of intervening *Alu *elements within FAP spacers permits identification of the boundaries of the FAP I:D imbalance phenomenon (Figure 2). The FAP I:D ratio as a function of *Alu *pair sequence number (APSN) are shown in Figure 3. Both unadjusted and CLIQUE-corrected I:D curves are provided in this figure. Figure 3A shows FAP I:D ratios across APSN values of ± 1,000 and reveals that the FAP I:D ratio depression appears to be limited to APSNs of ≤ 100. Further refinement of this I:D depression boundary was accomplished by grouping 10 consecutive APSNs together. This increased the FAP sample size from approximately 555,000 to over 5.5 million. The larger sample size improved the precision of detection of the I:D depression boundary to an APSN value of ± 107 (Methods).

Over 50 million FAPs reside within the CLIQUE-adjusted FAP I:D imbalance window. Based on the CLIQUE-adjusted I:D values illustrated in Figure 3, human direct FAPs outnumber inverted FAPs by 629,027 (Additional File 1, Table S1). Random variation reduces this difference to 613,924 (*P *< 0.05). Figure 3C magnifies Figure 3A to APSN values of ± 15 and illustrates that the greatest departure between CLIQUE-adjusted and unadjusted FAP I:D ratios occurs for APSNs of less than five. The largest APSN for a FAP residing within a single human CLIQUE is 0,31. Consequently, no CLIQUE adjustments to the FAP I:D ratio are required for APSN values greater than 31.

### PCR evidence of *Alu *pair exclusions in the chimpanzee genome

**Figure 4:**
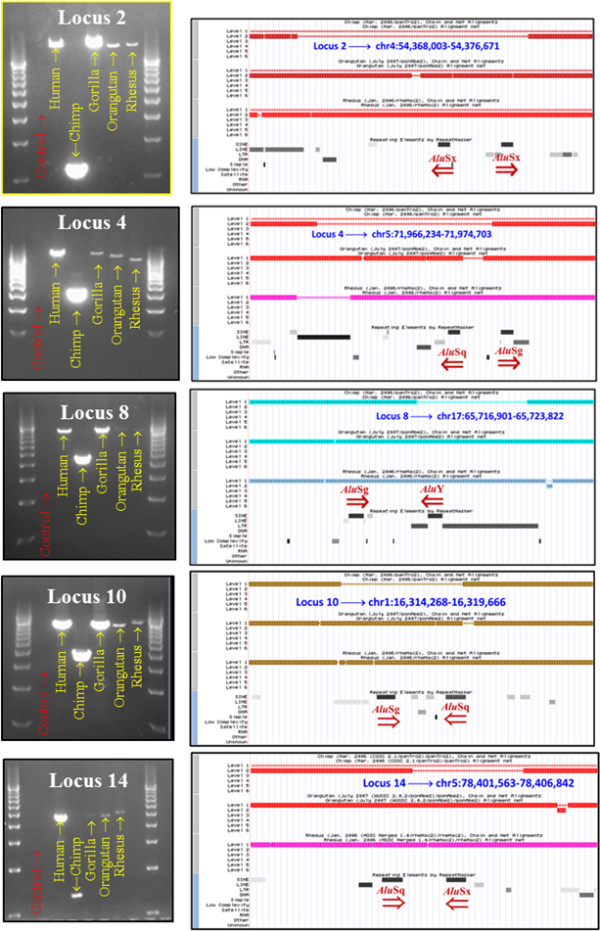
**Chimpanzee-specific APE deletions**. PCR analysis confirmed chimpanzee-specific APE deletions in orthologous human, chimpanzee, gorilla, orangutan and rhesus macaque loci. Human adjacent inverted FAP loci were chosen with spacer sizes between 651 and 1500 bp and a minimum of 1,000 bp of *Alu*-free flanking sequence. PCR loci were selected for which the chimpanzee loci were > 350 bp shorter than the human ortholog. Using identical primers, PCRs were then prepared for human, chimpanzee, gorilla, orangutan and rhesus macaque. APE: *Alu *pair exclusion; bp: base pair; FAP: full-length *Alu *pair; PCR: polymerase chain reaction.

We have presented computational evidence for a significant FAP I:D ratio imbalance in the human genome. To investigate our hypothesis that this imbalance may be due to the increased instability of inverted *Alu *pairs, resulting in APEs, we compared the human genome (hg18) to the chimpanzee genome (panTro2) to identify potential APE deletions. A total of 58 APE deletion candidate loci were identified for evaluation by PCR (Methods) in the chimpanzee genome through comparison of the human, chimpanzee, orangutan and rhesus macaque genome draft sequences. Fourteen of these loci were selected for PCR examination. These validations confirmed that 10 of these 14 loci had undergone chimpanzee-specific deletions consistent with inverted FAP instability. PCR primer design was problematic for the remaining four loci. No instances of false positive identification of chimpanzee-specific deletions were observed. The characteristics of the 10 loci confirmed as chimpanzee-specific deletions are summarized in Table 1. Images of gel chromatographs of the experimental interrogation of five of the loci are shown in Figure 4.

**Table 1:** Chimpanzee-specific APEs characterized by PCR

Locus ID	Position (hg18)	5' *Alu *Element			Spacer (bp)	3' *Alu *Element			Chimpanzee deletion Size (bp)
					
		Subfamily	Length (bp)	Strand		Subfamily	Length (bp)	Strand	
**1**	chr1:105842254-105848252	*AluY*	300	Positive	1,407	*AluJb*	291	Negative	4,896

**2**	chr4:54368003-54376671	*AluSx*	297	Negative	1,292	*AluSx*	310	Positive	5,829

**3**	chr2:68246922-68253405	*AluY*	312	Negative	1,237	*AluY*	304	Positive	3,413

**4**	chr5:71966234-71974703	*AluSq*	293	Negative	1,012	*AluSg*	310	Positive	4,307

**5**	chr13:64130795-64137788	*AluJo*	297	Positive	1,312	*AluSx*	292	Negative	2,776

**8**	chr17:65716901-65723822	*AluSg*	303	Positive	1,285	*AluY*	300	Negative	5,585

**9**	chr8:53032075-53037664	*AluSx*	309	Positive	973	*AluSx*	307	Negative	2,340

**10**	chr1:16314268-16319666	*AluSg*	296	Positive	793	*AluSq*	309	Negative	1,907

**14**	chr5:78401563-78406842	*AluSq*	313	Positive	665	*AluSx*	301	Negative	1,656

**15**	chr4:68494452-68500177	*AluY*	318	Negative	1,121	*AluSx*	286	Positive	1,654

APE: *Alu *pair exclusion; bp: base pair; PCR: polymerase chain reaction.

A secondary purpose of these PCR examinations was to assess the accuracy of the hg18 and panTro2 genome assemblies at loci involved in APE deletions. If we broadly assume that the combined hg18/panTro2 genome assemblies provide at least 50% accuracy in identification of inverted APE deletion loci, the probability of successfully validating five of these events in five consecutive PCR evaluations would be *P *= 0.03125 (0.5^5^). The fact that we were able to validate 10 such APE events in 10 consecutive PCR reactions with no evidence of false positives provides over 95% confidence that these two assemblies are at least 74 percent accurate (0.74^10 ^= 0.04924). When we compared the PCR-based estimate of chimpanzee-specific inverted APE deletions to the computationally derived estimate of human inverted APE deletions for this same data set, we found these results to be within 15 percent of each other (108 versus 94). The computation was based upon the human FAP I:D ratio (0.931) for loci satisfying the original PCR criteria (Methods). Thus, these data provide strong evidence for the existence of APE-induced genomic deletions. The characteristics of the 10 loci confirmed as chimpanzee-specific deletions are summarized in Additional File 1, Table S4. Images of gel chromatographs of the experimental interrogation of five of the loci are shown in Figure 4.

Chimpanzee-specific APE deletions within these (human) orthologous loci were estimated to have occurred during the six million years following the divergence between human and chimpanzee lineages [32].

### Comparison of orthologous human-chimpanzee direct and inverted FAP loci

An effort was made to better compare the characteristics of deletions within direct and inverted FAP loci. Loci selection criteria for this evaluation were identical to those used for PCR validation with two exceptions: direct FAP loci were included and chimpanzee loci were limited to those that were 1,000 to 2,000 bp shorter than their human ortholog. The second constraint was applied to avoid lengthy deletions that could be more difficult to analyze and to provide a reasonable sample size for manual analysis. Surprisingly, these criteria generated an almost equal number of shorter direct (193) and inverted (187) chimpanzee orthologs. A subsequent examination of the shorter direct chimpanzee FAP loci revealed that inverted APE-related deletions can plausibly be attributed to 93 (48%) of these shorter orthologous loci. These deletions are consistent with an interaction between a member of the direct FAP and a flanking *Alu *element in the opposite orientation. Furthermore, excluding chimpanzee orthologs that are shorter because of a human-specific retrotransposon insertion, fully 75 percent of the balance of the shorter chimpanzee loci can be plausibly attributed to have resulted from a flanking inverted APE-related deletion (see Methods and Additional File 1, Table S2). The attribution of shorter chimpanzee orthologs to possible inverted APE-related deletions is based upon the hypothesized APE deletion mechanism involving the resolution of *Alu*-induced double-strand breaks outlined in Additional File 1, Figure S7. This hypothesized APE deletion pattern applies to interactions between inverted FAPs with spacer sizes over 50 bp.

## Discussion

Non-random differences between direct and inverted FAPs exist for spacer sizes of zero to ≤ 350,000 bp. These differences may reflect orientation biases for either *Alu *element insertions or deletions. The instability of *Alu *pairs with spacer sizes below 650 bp has been previously described [26]. Our research suggests that additional mechanisms may be operational.

### APE mechanisms

**Figure 5:**
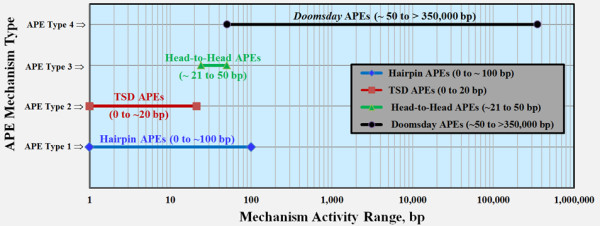
**Estimated ranges for four potential APE mechanisms for FAPs**. This semi-log chart illustrates the activity of the one previously identified [25] and three new APE mechanisms. The APE Type 1 mechanism can also be termed 'hairpin APEs' and has been previously identified as related to *Alu-Alu *hairpin formation with subsequent deletion. The range of this mechanism has been demonstrated to extend up to 100 bp in a yeast model [25]. The APE Type 2 mechanism can be described as 'TSDs APEs' and refers to a potential orientational insertion preference for *Alu *element insertions within the TSD of existing *Alu *elements. This mechanism would preferentially form direct-oriented FAPs. As with TSD APEs (Type 2), the Type 3 APE mechanism appears to reflect an insertional preference for the formation of head-to-head (inverted) FAPs. Type 3 APEs occur approximately within the range of 21 to 50 bp (Figure 1). The proposed mechanism for formation of Type 4 APEs is described in Figures 6 and S7 and is hypothesized to arise through a DNA conformation termed a 'doomsday junction'. APE: *Alu *pair exclusion; bp: base pair; FAP: full-length *Alu *pair.

Four separate mechanisms are theorized for generating APEs within the human genome (Figure 5). Although some overlap likely exists for the spacer size ranges wherein these four mechanisms operate, the first three mechanisms appear to be confined to adjacent FAPs that are separated by ≤ 100 bp. The first of these small-spacer APEs is identified by the observation that inverted *Alu *pairs form near-palindromic sequences that are vulnerable to hairpin formation and can induce double-strand breaks. This mechanism is termed 'hairpin APE' (Figure 5) and is thought to be operational between spacer sizes of 0 and approximately100 bp [25].

The second mechanism is termed 'TSD APE' and appears to be active for spacer lengths of less than 23 bp (Figure 1B). This spacer length only slightly exceeds the 7 to 20 bp size range for TSDs [2]. The nexus of high FAP density coupled with low I:D ratio is unique to human FAPs with these spacer lengths. The instability of inverted *Alu *pairs with spacer lengths of ≤ 100 bp has been demonstrated in a yeast model [25]. This instability would be expected to reduce the FAP I:D ratio. However, the coincident phenomena of high FAP density and low FAP I:D ratio may also be associated with the TPRT insertion mechanism. *Alu *elements inherently provide an increased density of L1EN target sites. These target sites are generated by *Alu *TSDs and by the adenine-rich region within *Alu *elements [36] (see also Additional File 1, *Alu-Alu *Insertions). The additional L1EN target sites coupled with *Alu *insertion bias associated with the RNA/DNA hybrid during the TPRT mechanism are consistent with the two superimposed patterns observed in Figure 1A. The instability of inverted *Alu *pairs almost certainly contributes to the low I:D ratios associated with closely spaced human FAPs. However, attribution of this instability to the entirety of the low I:D ratio observed for FAPs with spacer sizes of ≤ 20 bp may be an overestimate.

The third small-spacer APE mechanism is termed 'head-to-head APE' and involves the elevated frequency of head-to-head FAPs present between spacer sizes of 23 and 50 bp. This elevated frequency is more pronounced for spacer sizes between 25 and 35 bp and very pronounced for spacer sizes of 27 to 30 bp. Within the spacer range of 25 to 35 bp, head-to-head (inverted) FAPs outnumber either type of direct-oriented FAPs (Additional File 1, Figure S2). For spacer sizes of 27 to 30 bp, head-to-head FAPs actually outnumber the sum of both direct-oriented FAP pair types. If direct-oriented FAPs are relatively stable entities, this region of elevated head-to-head frequency may evidence an insertion-related phenomenon. A more detailed discussion of this possibility is provided in Additional File 1 (Possible Epigenetics Associated with Head-to-Head FAPs with Spacer Sizes of 24-36 bp).

**Figure 6:**
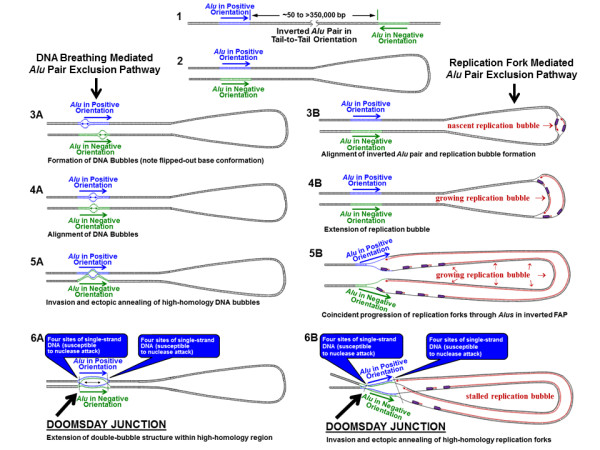
**Possible mechanisms for formation of G and S phase DDJ**. Steps 1 and 2 illustrate an inverted FAP. When the DNA in Step 1 is bent 180°, the two *Alu *elements within the inverted FAP are aligned. Steps 3A-6A and 3B-6B illustrate two possible mechanisms for interactions between inverted *Alu *elements without the formation of a hairpin loop. Steps 3A-6A, DNA Breathing (G phase) Mediated APE deletion. (3A) DNA breathing bubbles are typically < 20 bp [45] and are characterized by flipping of the unpaired nucleotide bases away from the center line of the double-helix [37]. A bubble in this conformation could be susceptible to interaction with a bubble of similar sequence. (4A) Simultaneous bubbles may arise in identical sections of aligned *Alu *elements. (5A) Simultaneous homologous bubble alignment could initiate bubble-bubble interaction with the potential for forming a 'double-bubble' conformation. (6A) The ectopic formation of the double-bubble conformation within two aligned breathing bubbles could potentially extend to the entire length of the two aligned *Alu *elements. The high GC content of *Alu *elements would likely increase the stability of the hypothesized doomsday junction. DDJs likely possess four single-stranded sections of single-stranded DNA at each end which could be susceptible to single-strand nuclease attack. Steps 3B-6B, Replication Fork (S phase) Mediated APE Deletion 3B-5B) Initiation and growth of a replication bubble. (5B) Coincident progression of the DNA replication bubble through an inverted FAP. (6B) Invasion and ectopic annealing of high-homology replication forks. APE: *Alu *pair exclusion; DDJ: doomsday junction; FAP: full-length *Alu *pair.

The fourth APE mechanism is very dissimilar from the first three small-spacer APE mechanisms in that it involves the loss of inverted FAPs separated by approximately 50 to ≤ 350,000 bp. The third APE mechanism overlaps this range up to a spacer size of 100 bp. Over 99 percent of all CLIQUE-corrected FAPs (not residing within the same CLIQUE) have spacer sizes greater than 100 bp. The higher energy state required for formation of single-stranded DNA makes hairpin loop formation a rare event between inverted *Alu *pairs separated by more than 100 bp [25,37]. Three possible pathways for interactions of distantly separated inverted FAPs are illustrated in Figure 6 and Additional File 1, Figure S7. Each of these pathways results in the ectopic annealing of single-stranded DNA associated with inverted FAPs. This annealing, which is hypothesized to result in a 'double-bubble' type structure, could potentially overcome the thermodynamic hurdle associated with single-stranded large-spacer hairpins. This structure is termed a 'doomsday junction' or DDJ (illustrated in Figure 6, Steps 6A and 6B and Additional File 1, Figure S7, Step 5).

Nuclease attack of DNA hairpins has been found to occur at the base, rather than the loop of DNA hairpins in yeast [23]. If DDJs exist, and if single-strand nucleases are active in primates, the eight single-stranded sections of DNA on the periphery of DDJs (Figure 6, Steps 6A and 6B and Additional File 1, Figure S7, Step 5) could form attractive nuclease targets. Such nicking could help resolve the DDJ. However, this nicking could potentially result in various combinations of flanking deletions on either side of the two *Alu *elements forming the DDJ. The resultant tell-tale deletion patterns that we would predict from this mechanism are outlined in Additional File 1, Figure S8. The varied repair products from nuclease attack on these single-stranded structures could result in partial or total removal of one or both *Alu *elements. These proposed patterns are consistent with those observed by PCR of possible chimpanzee-specific APE deletions shown in Figure 4 and Additional File 1, Figure S8D. The pattern is also consistent with deletion patterns in 199 of 380 orthologous human-chimpanzee FAP loci (51%) where a potential chimpanzee deletion had occurred (Additional File 1, Table S2). This deletion pattern increases to 75 percent when the 114 human-specific retrotransposon insertions are removed from the data set.

### G-phase doomsday APEs

Figure 6 and Additional File 1, Figure S7 outline separate mechanisms by which DDJs could form during the G and S phases of the cell cycle. We propose that G-phase DDJs result from the ectopic invasion and annealing of high-homology bubbles associated with DNA breathing (Figure 6, Steps 1-6A). Nucleosomes and other chromatin structures mitigate DNA breathing and thus may reduce the potential for G-phase DDJ formation. Therefore, in addition to their multifarious roles in signaling and protein binding, nucleosomes may also serve to minimize the interaction between high-homology DNA strands. The instability of closely spaced inverted *Alu *elements shown here and noted by previous researchers may be evidence that nucleosomes are either absent from hairpin prone DNA sequences or provide insufficient interference for hairpin formation [3,25,26]. The postulated G phase DDJ phenomenon may enjoy this same dominance over nucleosome interference.

If simultaneous DNA breathing bubbles were to arise between aligned homologous sequences, the flipped-out conformation of complimentary bases on both strands could provide additional potential for intra-strand interaction (Figure 6, Step 4A) [10]. This altered genomic structure formed by the hypothetical interaction between two homologous DNA bubbles would effectively create the double-bubble conformation associated with DDJs. The initial smaller double-bubble structure (Figure 6, Step 5A) could easily expand to form a larger double-bubble which could extend to almost the entire length of the two aligned *Alu *elements (Figure 6, Step 6A). The high GC content (> 60%) of *Alu *elements composing the large bubble conformation would likely enhance the stability of the hypothesized DDJ.

### S-phase doomsday APEs

S phase DDJs are proposed to result from invasion and subsequent annealing of high-homology DNA replication forks (Figure 6, Steps 1-6B and Additional File 1, Figure S4). Coincident passage of replication forks through inverted FAPs could provide an environment susceptible to formation of an S-phase DDJ. Unlike the chromatin interference present in G phase, replicating S-phase DNA is forced to lift its chromatin kimono and becomes much more vulnerable to ectopic DNA interaction. While single-strand binding proteins stabilize single-stranded portions of the replication fork, they are eventually displaced with a newly replicated strand of single-stranded DNA. This second strand could conceivably be supplied from an invading second replication fork.

Notably, upon formation of an S-phase DDJ, the DNA replication apparatus would be completely assembled and could potentially proceed, albeit in an ectopic fashion, and conceivably generate segmental duplications. In addition, the double-bubble binding of near-homologous *Alu *elements within a DDJ could invite the activity of cellular mismatch repair mechanisms. Such mismatch activity could help explain elevated mutation rates which have previously been observed close to deletions [38].

Finally, the DDJ mechanisms outlined in Figure 6 and Additional File 1, Figure S7 do not preclude interactions between direct-oriented FAPs. However, the distinctive 'V' shape of replication forks may provide steric hindrance to interactions with direct pairs and thus preferably favor interactions between inverted pairs. Regardless of the mechanism(s) associated with the human FAP I:D ratio imbalance, this metric is not an absolute measure of change in the number of either direct or inverted FAPs, but of the relative change between the two types.

## Conclusions

Direct and inverted FAPs are distributed non-randomly in the human genome. This non-random pattern exists for APSNs ≤ 107 bp and for spacer sizes up to 350,000 bp. A total of 59,357,435 FAPs (CLIQUE corrected) reside within this window and direct FAPs outnumber inverted FAPs by 629,027 (over two percent). Random variation only reduces this imbalance to 613,924 (*P *< 0.05). Outside of CLIQUEs, no known orientation insertion preferences exist for *Alu *elements. We believe that APE-related deletions may be responsible for a substantial proportion of the imbalance of over 600,000 between inverted and direct human FAPs. Future investigations of the APE phenomenon should better illuminate the mechanisms involved and characterize its extent in primate genomes.

## Methods

### Data acquisition and management

Data used in the research was obtained from the RepeatMasker [39] output for the hg18, 2006 Human Genome assembly. This data was downloaded from the UCSC genome BLAT Table Browser http://genome.ucsc.edu/cgi-bin/hgTables[40] and imported to Excel 2010 (Microsoft Corporation; Redmond, Washington). Orthologous chimpanzee, orangutan and rhesus macaque loci were obtained using the panTro2, ponAbe2 and rheMac2 genomes assemblies, respectively. Statistics were calculated using Minitab 15 (Minitab Inc.; State College, Pennsylvania).

### Histogram of human *Alu *size distribution

The RepeatMasker scan of the hg18 human genome assembly identifies potential *Alu *fragments as small as 12 bp. Using a haploid genome size of 3.1 × 109 bp, a total of 185 instances of a given 12 bp should randomly occur in human DNA. However, most *Alu *elements have sequence identities between 65 and 85 percent [26]. Using the lower sequence identity (65%) increases the number of random instances of a 12 bp target sequence occurring in the human genome from 185 to 32,485 (Additional File 1, Figure S1). The target sequence must increase in length to 26 bp before statistical significance (*P *< 0.05) occurs. This sequence size increases to 29 bp for 60% identity. For this study, only *Alu *sequences of ≥ 30 bp are used. For perspective, a 30 bp *Alu *fragment length is roughly 10 percent of the length of a full-length *Alu *element. Finally, it should be noted that the 12 bp sequences become significant (*P *< 0.05) when a segment of DNA shorter than 4,770 bp is being evaluated.

Sequences of less than 30 bp in length cannot be reliably determined to be actual *Alu *elements and are therefore excluded from this truncated percentage. A lower size limit of 275 bp is set to avoid I:D ratio directional bias caused by fragmented elements that can be generated by *Alu *insertions into a preexisting *Alu *element (Additional File 1). The upper *Alu *element size limit of 325 bp is set to avoid the potential for confounding results by inclusion of the smaller population of larger elements.

### Terminology for non-adjacent *Alu *pairs

The central *Alu *in this naming convention is always designated with the number '0'. The second member of the pair is designated by its sequential separation from the central *Alu*. If this second member of a pair is located 5' of the central *Alu *element, it is designated by a negative number and by a positive number if it is located 3' of the central *Alu *element. The value of the sequential separation of a given *Alu *element from the central *Alu *is defined as its APSN. For adjacent elements, these FAP pairs are described as -1,0 and 0,1. Similarly, FAPs separated by 25 intervening *Alu *elements are described as -26,0 and 0,26 pairs, respectively.

### Determination of 95% confidence interval for FAP I:D ratios

FAP sample sizes used in this study range from 555,354 to 567,242 (APSNs 0,1 to 0,107). These sample sizes are retrieved by counting functions within the *Alu *element Excel spreadsheet. Following removal of FAPs residing within the same CLIQUE (CLIQUE-adjusted), these data set sizes are reduced to between 460,588 and 557,364. CLIQUE-adjusted samples sizes below 550,000 only exist for APSNs ≤ 4. For a FAP sample size of 550,000, the number of direct and inverted FAPs should range between 274,272 and 275,728 (*P *< 0.05). Any imbalance in direct or inverted FAPs is offset by an equal and opposite imbalance in the other FAP type. Therefore, the I:D ratio for a sample size of 550,000 is expected to range from 0.9947 to 1.0053 (*P*≤ 0.05). This range increases to between 0.9942 and 1.0058 for the lowest sized (0,1) FAP family of 460,588.

### Determination of maximum APSN within the FAP I:D ratio imbalance window

Determination of the limits of the FAP I:D ratio imbalance boundary beyond an APSN of approximately 85 (Figure 3B) was accomplished by increasing the precision of the method. This added precision was achieved by increasing the FAP sample size. This larger sample size was acquired by calculating a 10-point moving average of the FAP I:D ratio across consecutive APSNs beyond the ± 85 range. This approach increased the FAP sample size from approximately 550,000 to 5.55 million and reduced the 95 percent confidence interval for randomness from 1 ± 0.0053 to 1 ± 0.0017. The highest ten consecutive APSNs which had an I:D average outside of these new confidence limits was the APSN range 103 to 112. The midpoint of this range is the APSN value of 107.

### Determination of maximum spacer size within the FAP I:D ratio imbalance window

Approximately 90 percent of the adjacent FAPs have spacer sizes below 6,400 bp. In addition, the I:D ratio for the upper 10 percent of this family is 0.9838 which is lower than the statistically significant I:D ratio of 1 ± 0.995. Consequently, determination of the boundary of the FAP I:D imbalance bubble (Figure 3B) requires examination of larger APSN families. The number of FAPs within a given size range can be summed across various APSNs. This summation was used to determine the spacer size boundaries for the FAP I:D imbalance window.

APSN families smaller than 0,25 contain very few members with spacer sizes between 300,000 and 400,000 bp. However, 3,541,238 FAPs reside within this spacer range for APSN's of 0,25 to 0,107. This spacer size range was divided into two separate ranges of 300,000 to 350,000 and 351,000 to 400,000. The number of FAPs within these spacer ranges was determined as 1,974,605 (I:D = 0.9951) and 1,566,633 (I:D = 0.9956), respectively. The expected ranges for FAP I:D ratios for these two spacer size ranges are 0.9972 to 1.0028 and 0.9969 to 1.0031, respectively (*P *< 0.05). These two I:D ratios are outside of these ranges and thus show that the FAP I:D imbalance window extends beyond ± 350,000 bp.

### Selection of loci for validation of APE deletions in the chimpanzee genome

The methodology employed for selection of potential APE deletion loci utilized five criteria. These criteria were pair orientation, APSN, *Alu *element size, spacer size and *Alu*-free flanking sequence 5' and 3' of the pair being evaluated. Only inverted *Alu *pairs were chosen as potential experimental loci as they have been previously demonstrated to be unstable [25]. The second criterion, APSN, was limited to 0,1 (adjacent) FAPs as any intervening *Alu *element necessarily forms a second, more closely spaced inverted pair with one of the two elements of that FAP. Therefore, any deletion associated with this locus could reasonably be attributed to interactions associated with the intervening element. For this reason, only the pool of adjacent human FAPs (APSN = 0,1) was used to identify candidate APE deletion loci.

The third criterion, *Alu *element size, was limited to the 275 to 325 bp constraints set for FAPs. The fourth criterion, spacer length separating the two FAP elements, was limited to those elements separated by 651 to 1,500 bp. The lower spacer size limit was set by the upper limit of previous work [26] and upper limit was set to provide an acceptable number of candidate loci. The fifth criterion, 5' and 3' *Alu*-free flanking sequence around a 0,1 FAP, was set to a minimum of 1,000 bp. This constraint was necessary to avoid attribution of an APE deletion to nearby elements. These criteria created locus sizes between 3,201 and 4,150 bp.

A total of 13,664 human loci were identified which satisfied these five criteria. This sample size was approximately 0.03 percent of the approximately 50 million CLIQUE-adjusted FAPs within the I:D imbalance window shown in Figure 3B. These loci were then compared to the chimpanzee panTro2 genome assembly using the LiftOver feature of the USCS Genome Browser [40-42]. This screening identified 715 (or slightly over five percent) of the chimpanzee loci that were over 350 bp smaller than their human ortholog. The less than 350 bp lower limit was set to reduce the number of false-positive loci (in other words, human specific *Alu *insertions can be flagged as potential sites for chimpanzee APE-related deletions). The 715 loci were individually inspected using the UCSC genome browser for the human, chimpanzee, orangutan and rhesus macaque genomes [40-44]. These inspections reduced the number of PCR candidate loci to 58. Four criteria accounted for approximately 90 percent of this reduction. These four criteria, in order of magnitude, were:

1. The presence of N's in the chimpanzee genome assembly (382 loci)

2. The insertion of a human specific transposable element as the cause of the smaller chimpanzee loci (141 loci)

3. A deletion present, but so large that it encompassed an adjacent *Alu *element making the deletion non-diagnostic (56 loci)

4. Complementary deletions were also present in orangutan or rhesus (38 loci)

The remaining 58 loci were selected as potential candidates for further examination with PCR.

### Estimation of APE deletions in chimpanzee genome by observation

Although only 58 of the 715 loci were accepted for further examination by PCR, an additional 94 of these loci showed considerable evidence of being potential APE deletions (criterions 3 and 4, above). Adding these 94 loci to the 58 PCR candidate loci increases the number of APE-related deletion loci to 152. It was also assumed that the 382 loci which contained N's in the chimpanzee (rejection criterion 1) were indeterminate and could neither be accepted nor rejected regarding detection of APE-related deletions. Separating these 382 loci (which contained N's in the chimpanzee deletion) from the original set of 715 loci reduces the total number of individually inspected loci to 333. It is estimated that 152 likely APE-related deletion loci exist out of these 333 loci (45.6%). Of the 14 loci evaluated by PCR, 10 were informative (71.4%). The PCR results from the remaining four loci were uninformative and no false positive instances of chimpanzee-specific deletions were observed. Combining these two probabilities provides an estimate that 32.6 percent (108) of the 333 loci were likely APE-related deletions. Therefore, within these 13,664 inverted FAP loci, a total of 108 APE-type deletions are estimated to have occurred in chimpanzee (by observation) since the human-chimpanzee divergence.

### Primer design for PCR

Candidate PCR amplicon sequences were obtained with the BLAT feature of the UCSC genome browser [40,42]. These sequences were aligned using the BioLign software (developed by Tom Hall and available from the Buckler Lab website: http://www2.maizegenetics.net/bioinformatics). These alignments were manually inspected for common identity between the four primate species. Forward and reverse oligonucleotide primers were selected from regions of common alignment. Primer sequences are shown in Additional File 1, Table S2.

### PCR amplification

All PCR amplifications were conducted in 27.5 μL reactions using 25 ng DNA template, 0.2 μM oligonucleotide primer, 1.25 units TaKaRa LA Taq™, 0.4 mM dNTPs, and 1X TaKaRa LA Taq™ buffer containing 2.3 uM MgCl_2_. A list of primers is provided in Additional File 1, Table S2. The primate panel contained templates from *Homo sapiens *(HeLa; cell line ATTCC CCL-2); *Pan troglodytes *(common chimpanzee "Clint", cell line Coriell Cell repositories NS06006), *Gorilla gorilla *(Western lowland gorilla; cell line Coriell Cell Repositories NG05251); *Pongo abelii *(Sumatran orangutan; cell line Coriell Cell Repositories NG06209); and *Macaca mulatta *(rhesus macaque; cell line Coriell cell Repositories NG07110). PCRs were run for 80 sec for initial denaturation at 94°C. Denaturing, annealing and extension times and temperatures were 20 sec at 94°C, 20 sec at optimum temperatures (Additional File 1, Table S2) and 8 min 30 sec at 68°C, respectively, for 32 cycles. The 32 cycles were followed by a final extension time of 10 min at 68°C. Following amplification, all PCR products were electrophoresed on 1.5% agarose gels stained with ethidium bromide at a concentration of 1 μl per 50 mL of gel solution. Gels were run for 45 to 55 min at 175 volts. Finally, fragments were visualized using UV fluorescence.

### Comparison of APE deletions in chimpanzee genome by computation and observation

Using the original criteria for isolating potential experimental loci, 13,664 inverted FAP and 14,680 direct FAPs were identified. The I:D ratio for these FAPs is 0.931 and the difference between these inverted and direct FAPs is 1,016, which we believe correspond to APE-associated deletion events. All *Alu *element insertions have occurred over the 65 million years of primate evolution. It is estimated that the most recent common ancestor of humans and chimpanzees lived approximately six million years ago [32]. Consequently, approximately 12 million years of genome evolution are estimated to have occurred between extant humans and chimpanzees. For this 12-million year period of evolution to be incorporated into calculated APE rate estimates, both orthologous chimpanzee-specific and human-specific APE-related deletions must be estimated. Only chimpanzee-specific APE-related deletions are measured in this study. Therefore, only half of the 12-million years of evolution are used (six million years) in this estimate. Therefore, a conservative estimate of 94 chimpanzee-specific APE deletions would be expected over the 6 million years since the human-chimpanzee divergence (1016 × 6 ÷ 65 = 94). This number is concordant with the 108 APE deletions previously estimated to have occurred by observational methods (see Estimation of APE Deletions in Chimpanzee Genome by Observation above).

### Moving average distributions of actual and random *Alu *clustering

The RepeatMasker scan of the hg18 human chromosome assembly recovers 102,592 *Alu *elements in chromosome 1. Since orientational clustering bias has been shown to occur within CLIQUEs, only the 5' *Alu *element in each CLIQUE was included in this evaluation. Chromosome 1 contains 50,262 *Alu *elements that do not reside within a CLIQUE. Human chromosome 1 contains 34,916 CLIQUEs, of which 26,277 contain at least one *Alu *element. Consequently, only 76,539 (50,262 + 26,277) *Alu *elements were used in this clustering evaluation. A value of +1 was assigned to each *Alu *on the positive strand and a value of -1 was assigned to each *Alu *on the negative strand. Moving average data was calculated for the 50, 100, 200, 500 and 1,000 sequential directional data points in Excel.

Five sets of 76,539 random +1 and -1 data (equivalent to the revised data set of *Alu *elements in human chromosome 1, above) were generated using Minitab15. This data was transferred to Excel and moving averages were calculated for each set of random data for 50, 100, 200, 500, 1,000, 2,000, 5,000 and 10,000 sequential directional data points. These 48 sets of moving average data (one set of actual data and five sets of random data for eight separate moving averages) were then transferred back to Minitab. Individual mean and standard deviations for each set of random distributions were determined using the Mintab15 histogram 'with fit and groups' algorithm. The five individual means and standard deviations were then averaged for each set of random moving averages. The random data curves were generated using these average mean and standard deviations (Additional File 1, Figure S3).

## Abbreviations

APE: *Alu *pair exclusion; APSN: *Alu *pair sequence number; bp: base pair; CLIQUE: catenated LINE1 endonuclease induced queue of uninterrupted *Alu*, LINE1 and SVA elements; DDJ: doomsday junction; dNTP: deoxyribonucleotide triphosphate; FAP: full-length *Alu *pair; I:D Ratio: ratio between inverted and direct *Alu *pairs; LINE1: long interspersed element 1; L1EN: endonuclease domain of LINE1 ORF2 protein; L1RT: reverse transcriptase domain of LINE1 ORF2 protein; LTR: long terminal repeat; ORF: open reading frame; PCR: polymerase chain reaction; SINE: short interspersed elements; SVA: SINE/variable number of tandem repeats/*Alu*; TPRT: target primed reverse transcription; TSD: target site duplication; See also Additional File 2.

## Competing interests

The authors declare that they have no competing interests.

## Authors' contributions

GWC, MKK, JAW and KH designed the research; GWC and JDM performed the research; MAB contributed new reagents and analytic tools; GWC analyzed the data and wrote the paper. All authors read and approved the final manuscript.

## Supplementary Material

Additional file 1 **Supplemental Information**. This file contains fundamental background information related to *Alu *pair exclusion research. It also contains discussions and data that support the findings within the manuscript. Click here for file

Additional file 2 **Definition of Terms**. This file contains a list with definitions of abbreviations and novel terminology introduced within the manuscript. Click here for file
