# Supplementary material for: Alu pair exclusions in the human genome
Source: Mob DNA. 2011 Sep 23;2:10. doi: 10.1186/1759-8753-2-10 (PMC3215922; doi:10.1186/1759-8753-2-10)
Supplement: Additional file 1 — Supplemental Information. This file contains fundamental background information related to Alu pair exclusion research. It also contains discussions and data that support the findings within the manuscript. [file 1759-8753-2-10-S1.DOCX]

**Supplemental Information**

**Table of Contents**

- *Alu-Alu* insertions, AAIs S2
- Orientational clustering of *Alu* elements – human chromosome 1(Figure S1) S2
- Catenated *Alu* clusters, CACs (Figure S2) S3
- *Alu* element fragment analysis S4
- Imbalance between the positive and negative oriented full-length *Alu* elements S5
- Possible epigenetics associated with head-to-head FAPs with spacer size of 24-36 bp S6
- Comparison of direct and inverted FAPs in orthologous human/chimpanzee loci (Table S1) S8
- Potential for ARMD masking of APE deletions (Figure S7) S9
- Figure S1 - Size distribution of *Alu* elements in the human genome S12
- Figure S2 - four Types of *Alu* pairs S13
- Figure S3 – directional *Alu* element clustering in human chromosome 1 S14
- Figure S4 – Size distribution of human catenated *Alu* clusters, CACs S16
- Figure S5 - The 1,000 most retrotransposon-rich CLIQUEs and the 1,000 longest CLIQUEs S17
- Figure S6 – CLIQUE adjusted adjacent FAP I:D ratio versus spacer size S18
- Figure S7 - Possible S phase two-bubble *Alu* pair exclusion (APE) pathway S19
- Figure S8 - Possible deletion patterns resulting from resolution of doomsday junctions S21
- Figure S9 - Proximity of nearest inverted human orthologous *Alu* element to chimp ARMDs S22
- Table S1 – Characteristics of CLIQUEs in hg18 S23
- Table S2 – CLIQUE-adjusted full-length *Alu* pair type sample sizes S24
- Table S3 - Comparison of orthologous direct and inverted FAP loci S25
- Table S4 – Primers for orthologous APE loci identified in Table 2 S26
- References S27

***Alu-Alu* insertions, AAIs**

The interruption of an older *Alu* element sequence by a younger *Alu* insertion has been observed by other researchers (Giordano et al. 2007). These interruptions are referred to as *Alu-Alu* insertions or AAIs. When an AAI occurs, the younger *Alu* element is flanked on its 5’ and 3’ ends by the left and right monomers of the older *Alu* element. In addition to counting the younger AAI insertion, this study treats each flanking monomer of the older *Alu* element as a separate element. This identification of two *Alu* elements as three, creates a natural bias of lowering the I:D ratio. The use of only full-length *Alu* pairs, FAPs, eliminates orientational AAI bias from the analysis.

Finally, while a characteristic of AAIs is their contiguity to other *Alu* elements, an estimated five percent of the fragmented *Alu* population (< 275 bp) is separated by over 500 bp from other *Alu*, LINE1 and SVA elements. The characteristics of these isolated and truncated *Alu* elements, isotruncs, may suggest past genomic instability (Supplemental Information, *Alu* Element Fragment Analysis).

**Orientational clustering of *Alu* elements – human chromosome one**

Figure 3 illustrates the FAP I:D imbalance across the entire human, hg18 genome assembly. It was postulated that this I:D bias should also be evident across smaller regions of the genome. Human chromosome one, chr1, was chosen to test this hypothesis. The purpose of this exercise was two-fold. The first purpose was to determine if directional clustering associated with *Alu* elements was greater than that which would be expected from random data. The second purpose was to determine what size group of *Alu* elements gave the largest departure from orientational randomness. It was determined that maximum *Alu* orientational clustering (CLIQUE-corrected) occurred for group sizes of 100-200 and 5,000-10,000 elements (Figure S3B). The orientational clustering within the smaller sized groups (100-200 elements) is consistent with that observed in Figure 3. Group sizes within these two size ranges demonstrated more than 40 percent greater orientational variation than that which is expected from random orientation (Methods).

**Catenated *Alu* clusters, CACs**

The TPRT mediated insertion of *Alu* elements typically results in the formation of target site duplications, TSDs. During this process, the original L1EN target site is partially duplicated on the 5’ end of the element and completely duplicated on the 3’ end of the element. In addition to this partial duplication, a third L1EN target site is also associated with new *Alu* insertions. This third target site is the adenine-rich sequence which separates the right and left monomers of full-length *Alu* elements. Slightly over one percent of all human *Alu* insertions occur within other *Alu* elements and the vast majority of these insertions occur within the adenine-rich spacer separating the two monomeric subunits[[1](#_ENREF_1)]. Therefore, in addition to preserving the original L1EN target site, each TPRT insertion of a full-length *Alu* element adds two additional L1EN target sites to the human genome. This clustering of L1EN target sites may serve as the catalyst for the observed clustering of *Alu* elements.

This tendency for *Alu* clustering may explain the 38,753 FAPs (6.9%) which have inserted within the TSD <20 bp size limits of a second *Alu* element. Figure 5 shows that two additional FAP I:D ratio biasing mechanisms appear to exist for adjacent FAPs separated by <50 bp. For purposes of this discussion, groups of *Alu* elements separated by <50 bp are described as catenated *Alu* clusters or CACs. Over 113,000 CACs reside in the human genome. The size distribution for human CACs is illustrated in Figure S4. Approximately 22% of adjacent FAPs fall within the CAC definition.

***Alu* element fragment analysis**

As can be seen in Figure S1, over 25 percent of the human *Alu* element population is less than full-length. Several mechanisms could be responsible for *Alu* fragmentation including internal insertions of one element into another (AAIs), partial deletions associated with unstable closely spaced inverted FAPs [[2](#_ENREF_2)] and non-canonical insertions[[3](#_ENREF_3)]. AAIs are characterized by their proximity to other *Alu* elements. However, deletions associated with closely spaced inverted FAPs and non-canonical insertions can occur in regions separated from other retrotransposons.

An analysis of human chromosome one reveals that the slightly over one-fifth of the *Alu* element population exists as truncated fragments with lengths of 200 bp or less. Some 2,400 of these truncated elements are present in chromosome one (~12 percent of the truncated population) and are separated from any LINE, SVA or other *Alu* element by at least 500 bp. These three retrotransposon families are known to be active in humans [[4](#_ENREF_4)] and the insertion of these active elements within the TSD of an existing *Alu* element could potentially result in some kind of fragmentation event. For purposes of this analysis, these isolated and truncated *Alu* elements are referred to as isotruncs. An inspection of 50 randomly chosen loci from among the 2,400 element data set identified two-thirds (33 of 50) as ancient left and right *Alu* monomers (FRAMs and FLAMs). The origin of these monomers predates *Alu* element formation and, consequently, these events were removed from the data set. Approximately one-third of the remaining loci (15 of 50) were identified as being actual *Alu* isotruncs. The remaining two elements in this fifty loci data set are immediately adjacent to an endogenous retrovirus and therefore may have been truncated by the endogenous retrovirus insertion. These two elements were excluded from further analysis.

From this analysis of human chromosome one, roughly one-half percent (624 estimated events out of 102,592 *Alu* elements in chr1) of human *Alu* elements exist as isotruncs. Isotruncs also comprise an estimated five percent of the population of non-contiguous (i.e., non-CLIQUE) fragmented *Alu* elements. Their isolation raises the possibility that underlying mechanism(s) forming these isotruncs may not be immediately attributable to retrotransposon insertions. The 500 bp flanking regions of the 13 isotruncs identified, above, were examined for homology with other regions of the human genome. The purpose of this examination was to determine if any of these regions could have been generated as part of a nearby retrotransposon insertion because of the potential for read-through of a weak termination signal during transcription [[5](#_ENREF_5), [6](#_ENREF_6)]. More specifically, could these regions of the genome have been originally transcribed along with a retrotransposon and have accompanied its eventual insertion? If true, the presence of an *Alu* isotrunc could be an artifact of such an unusual retrotransposon insertion event. Excluding the homology contributions of dormant SINE elements within the flanking regions, all but one of the isotruncs had low homology (< 15% of the 500 bp sequence) with other portions of the human genome. While far from conclusive, this evidence is consistent with the model that truncated *Alu* elements could have been generated from deletion events.

**Imbalance between the positive and negative full-length *Alu* elements**

The departure from unity in the I:D ratio for adjacent FAPs is, in part, the result of a non-random imbalance between positive and negative orientations for full-length human *Alu* elements. The 806,880 full-length human *Alu* elements do not appear to be randomly distributed with respect to orientation. The orientational breakdown of this population is 49.80% in the positive and 50.20% in the negative orientation, respectively (p = 0.0044). This distribution would be expected to fall within 49.89% to 50.11% for a random distribution (p = 0.05). It should be noted that the human adjacent FAP population is less than the full-length *Alu* element population (560,485 and 860,880, respectively). The adjacent FAP population is smaller than the full-length *Alu* element population because of the interspersion of fragmented *Alu* elements (<275 bp) within the full-length *Alu* population.

The insertional bias associated with full-length *Alu* elements appears to affect only clustered *Al*u elements. Removal of clustered elements (more detail, below) from the full-length *Alu* element data set returns the positive/negative orientation ratio to a range that would be expected with random insertions. There are 442,187 non-clustered adjacent human FAPs. The fraction of positive and negative oriented *Alu* elements within this group is 49.90% and 50.10%, respectively (p=0.22).

**Possible epigenetics associated with head-to-head FAPs with spacer size of 24-36 bp**

Head-to-head FAP frequencies are elevated within the spacer size range of 24-50 bp (Figure 1B). More notable is that this FAP frequency exceeds each type of direct oriented FAPs between spacer sizes of 25-35 bp. It is intriguing that *Alu* insertions within *Alu* TSDs [[4](#_ENREF_4)] predominantly form direct FAPs and yet appear to form inverted FAPs when spacer sizes are between of 24 and 36 bp. Assuming that direct FAPs are reasonably stable entities, the latter may be evidence of a previously-uncharacterized inverted *Alu* insertion mechanism.

One explanation for this pattern is that nucleosomes may be attracted to head-to-head FAPs with spacer sizes of 24-36 bp. However, this theory does not explain why head-to-head FAP frequencies within this spacer range exceed the number of either type of direct-oriented FAPs. The fact that head-to-head FAPs within this spacer size range actually exceed either type of direct-oriented FAP may indicate that an insertional mechanism is driving this phenomenon. A second explanation for this pattern of elevated head-to-head FAPs is that L1EN may somehow associate with the 5’ end of *Alu* elements. In addition to this association, the mechanism would also require L1EN to cleave its target sequence on the sense strand, approximately 24-36 bp from the 5’ end of an existing *Alu* element. This orientational nicking, coupled with subsequent formation of the TPRT PolyA/PolyT, RNA/DNA hybrid would drive orientation of the new FAP toward the head-to-head orientation.

The GC content of the human genome has been estimated to be 41 percent [[7](#_ENREF_7)] With this GC frequency, the probability of the 5’-TTTTAA-3’ L1EN target sequence randomly centering at any locus is one chance in 1,517. With the 806,880 full-length *Alu* elements in the human genome, this target site should randomly occur 6,914 times within the 24-36 bp spacer span for high head-to-head FAPs. The actual number of human head-to-head FAPs possessing spacer sizes within this range is 3,464. This actual number is 50.1 percent of the theoretical 6,914 L1EN target sites that are predicted to be centered randomly within this same 24-36 bp range. The highest incidence of head-to-head FAPs is 74 percent of the theoretical estimate which occurs at a spacer size of 28 bp. Some flexing of DNA between the L1EN anchoring site and cut site could possibly explain the high incidence of head-to-head FAPs spanning across the 13 nucleotides within the 24-36 bp spacer range.

The genetic distance of a 28 bp spacer size is equivalent to approximately three turns of DNA or about 100 Å (in non-bent conformation). The physical size of L1EN is approximately 25 bp, or 80 Å [[8](#_ENREF_8)]. Possibilities for an L1EN association with the 5’ end of an *Alu* sequence include 1) direct L1EN binding with DNA flexing, 2) indirect L1EN association through a scaffolding protein, or possibly 3) direct L1EN binding plus dimerization because of the proximity of the two *Alu* elements in the head-to-head FAP orientation. The sustained presence of L1EN and any associated proteins could also inhibit inverted *Alu* pair instability previously noted by other researchers [[9](#_ENREF_9)].

**Comparison of direct and inverted FAPs in orthologous chimpanzee/human loci**

The identification of a non-CLIQUE human FAP I:D imbalance has been identified by computational analyses. This work is complemented by PCR evidence of selected loci. Further examination into the APE phenomenon was made by examination of orthologous direct and inverted FAP loci in the chimpanzee genome (panTro2) and the human genome (hg18). The results of this examination are shown in Table S3. As with PCR comparisons (Figure 4), the selection criteria for these FAP loci were a spacer size of 651-1,500 bp with 1,000 bp of *Alu*-free flanking sequence. Once identified, these initial loci were filtered using the LiftOver feature in BLAT. All chimpanzee loci which were 1,000 -2,000 bp shorter than their human orthologue were chosen for manual examination.

The total direct and inverted FAP loci selected for individual examination were 193 and 186 loci, respectively. Evidence for shorter chimpanzee sequences fell into three categories; A) human specific retrotransposon insertion or repetitive DNA insertions (116 loci), B) possible APE-related deletions (254 loci) and C) possible non-*Alu* inverted sequence deletions (8 loci). The focus of this examination was category B. Category B is further broken down into three sub-categories. The first sub-category (201 direct plus inverted loci) contains an orthologous inverted FAP which can be reasonably associated with an APE-related deletion in chimpanzee. The second sub-category (53 direct plus inverted loci) contains an unexplained indel. Each of these 53 loci were found to contain (within the human indel) at least one consensus L1EN target sequence in the orientation required to form an inverted *Alu* pair. In the case of this second subcategory, the insertion of a chimpanzee-specific *Alu* element within the indel could potentially generate an inverted APE deletion event. Such an *Alu* insertion would have the potential to eliminate the new *Alu* insertion from detection.

An unexpected finding in the third sub-category of Table S3 was the presence of a perfect inverted sequence (from 7 to 22 bp) separated by a spacer within the human indel. This self-contained inverted sequence could potentially create inherent genomic instability within the indel sequence (Lewis et al. 1999). This inverted sequence could also be subject to genomic interactions similar to those proposed in Figures 6 and S7. Summing subcategories one and two, the potential fraction of APE deletions in these direct versus inverted FAP loci was 60.6 percent and 73.7 percent, respectively.

**Potential for ARMD masking of APE deletions**

One of the patterns which may be associated with an inverted APE-related deletion can be generated by a DNA double-strand break repair process known as single-strand annealing, SSA. SSA, which utilizes high-homology direct repeats as a repair template, can create a repair pattern that mimics an intra-chromosomal slippage and recombination event. Direct-oriented *Alu* elements in the vicinity of an inverted APE-related deletion could possibly be used as templates in the SSA repair process [[10](#_ENREF_10)]. APE deletions which are repaired by SSA could produce a chimeric *Alu* element which would appear as *Alu* recombination mediated deletions, ARMDs[[11](#_ENREF_11), [12](#_ENREF_12)] It is interesting to note from Table S2 that 16 percent of the direct FAP loci that were identified as possible APE deletions were also associated with an ARMD pattern of repair. Similar inverted loci had five percent of deletions associated with the ARMD pattern of repair. It is not possible to determine whether these ARMDs were formed by inter-chromosomal slippage/recombination or SSA associated with an unknown deletion. The 3X disparity in the percentage of ARMDs between direct and inverted APEs appears to be attributable to the opportunity to form ARMDs between the members of direct FAPs that is absent in the inverted FAP loci. An examination of the 15 ARMDs associated with direct loci showed that ten were associated with the originally identified direct FAP and five were associated with *Alu* elements flanking the direct pair. Thus, the number of flanking ARMD repairs (ARMDs between one element in the target FAP and a second flanking *Alu* of identical orientation) was identical for both inverted and direct loci.

ARMDs occasionally skip over one or more *Alu* elements before recombining with another *Alu* element [[12](#_ENREF_12)] This same *Alu* skipping feature could potentially be associated with an APE-deletion model followed by SSA repair. Unfortunately, SSA destroys the evidence of the original source of a deletion. Therefore, the possibility of SSA repair following an inverted APE-deletion cannot be eliminated as a possible cause of ARMDs.

An attempt was made to evaluate chimpanzee ARMDs as potential APE loci. This was accomplished by evaluating ARMD loci from previous work (Han et al. 2007). The first 100 chimpanzee ARMD loci were evaluated for their closest proximity to an inverted *Alu* element. A histogram of these distances is shown in Figure S9A. This figure shows that 95 percent of these ARMD loci contain an inverted full-length *Alu* element within 8,500 bp of one of the chimeric elements composing the ARMD. All of the 100 loci fell within 25,000 bp of an inverted element. The 25,000 bp span of these ARMDs closely matched the range of the APSN5 FAP family. Figure S9B is a linear regression of the I:D ratio across the ten spacer percentiles of this family. The total, CLIQUE adjusted, population for the APSN5 family is 551,764 FAPs. Each percentile contains slightly over 50,000 data points and provides a 95 percent confidence interval of ± 1.7 percent from unity (green dashed line in Figure S9B). All of these ARMDs fall outside the range of this confidence interval, indicating that APE deletions followed by SSA between direct *Alu* pairs may therefore be considered as one possible mechanism for the formation of ARMDs.

**Figure S1**

**
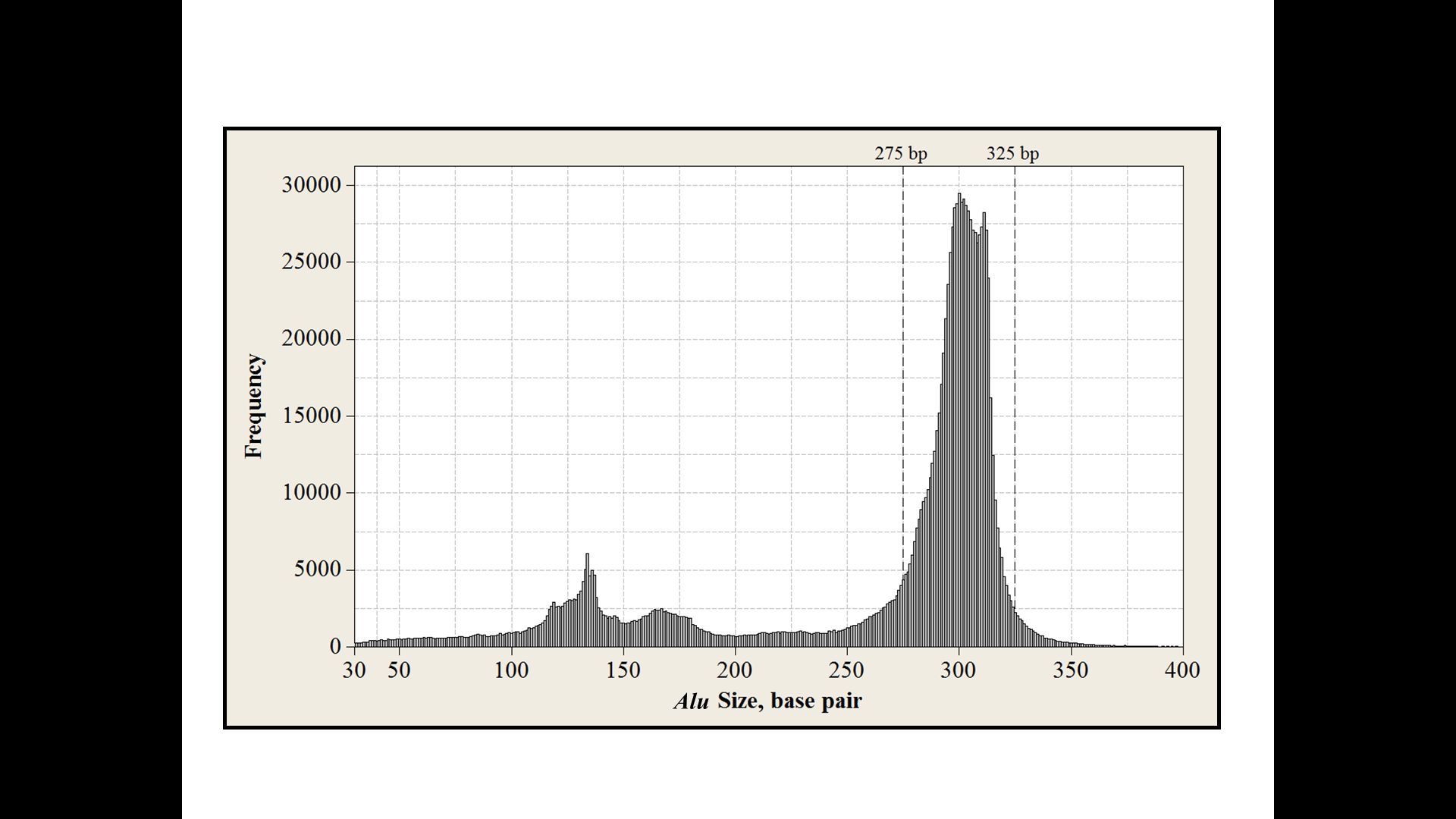
Figure S1. Size Distribution of *Alu* elements in the Human Genome.** A total of 1,172,576 *Alu* elements (non-random) are present in the RepeatMasker scan of the hg18 genome assembly. Approximately 29.0% of these *Alu* elements have lengths less than 275 bp, 68.8% have lengths between 275 bp and 325 bp, and 2.2% have lengths greater than 325 bp. The lower limit of 30 bp is set by certainty that a given sequence is an actual *Alu* element (p<0.05).

**
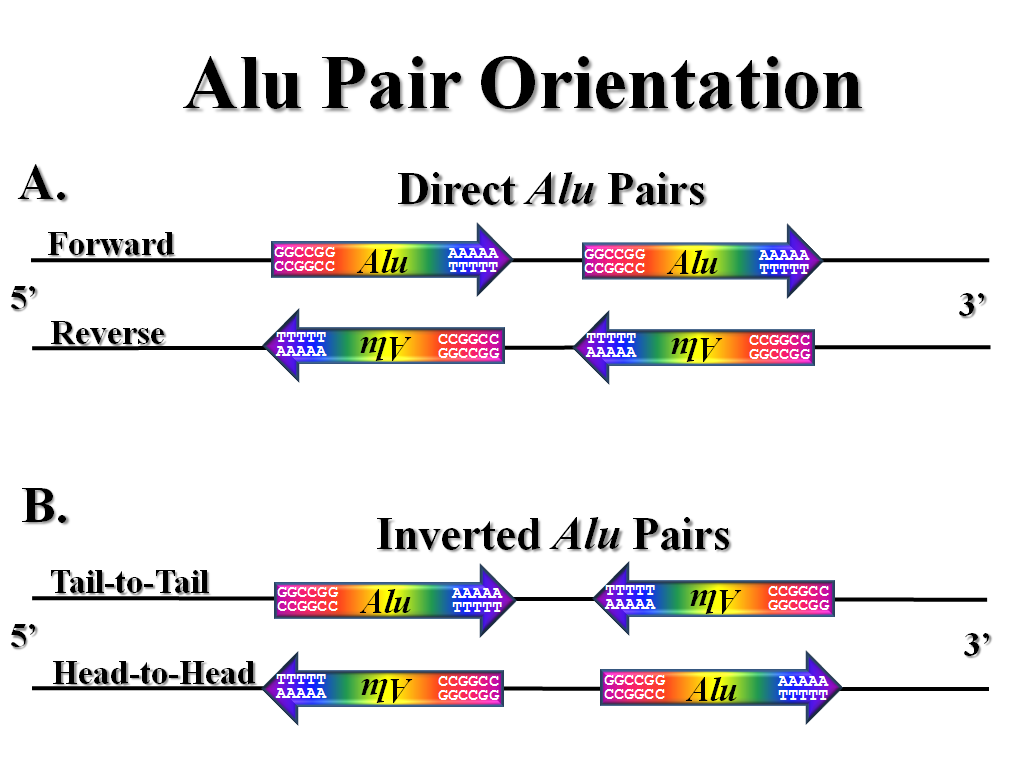
Figure S2**

**
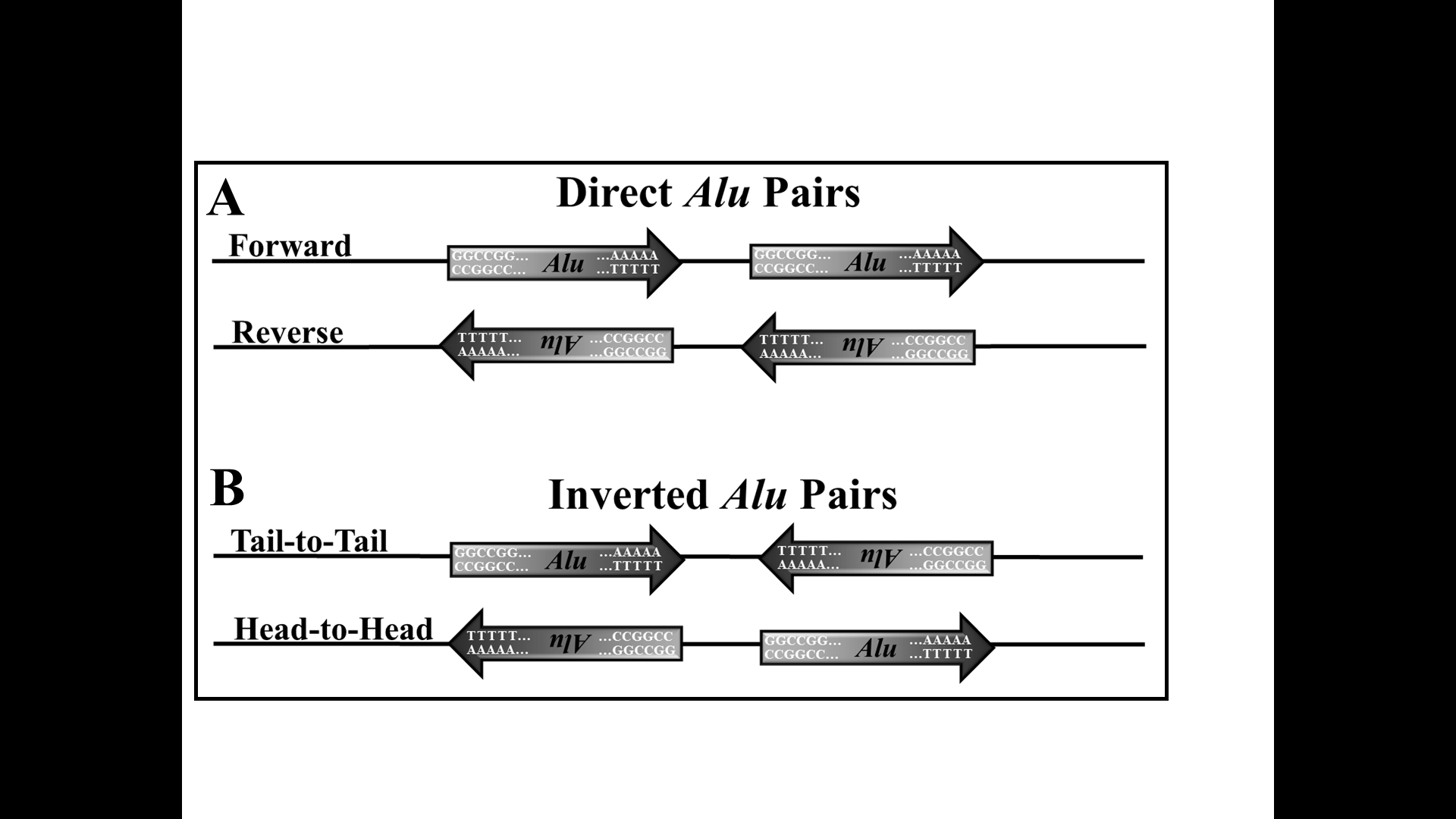
**

**Figure S2. Four Types of *Alu* Pairs.** Because of the directionality of *Alu* elements, four orientational combinations are possible for *Alu* pairs. A. Direct *Alu* pairs exist when both elements are in the same orientation. When each *Alu* element is in the positive direction, the pair is defined as being in the “Forward” orientation. When both *Alu* elements in the pair are in negative orientation, the pair is defined as being in the “Reverse“ orientation. B. Inverted *Alu* pairs are defined as those pairs which have the two elements in opposite orientations. When an inverted *Alu* pair is oriented with the poly(A) tails pointing toward each other, the pair is defined as being in the “Tail-to-Tail” orientation and when an inverted pair is oriented with the poly(A) tails pointing away from each other, it is defined as being in the “Head-to-Head” orientation.

**Figure S3**

**
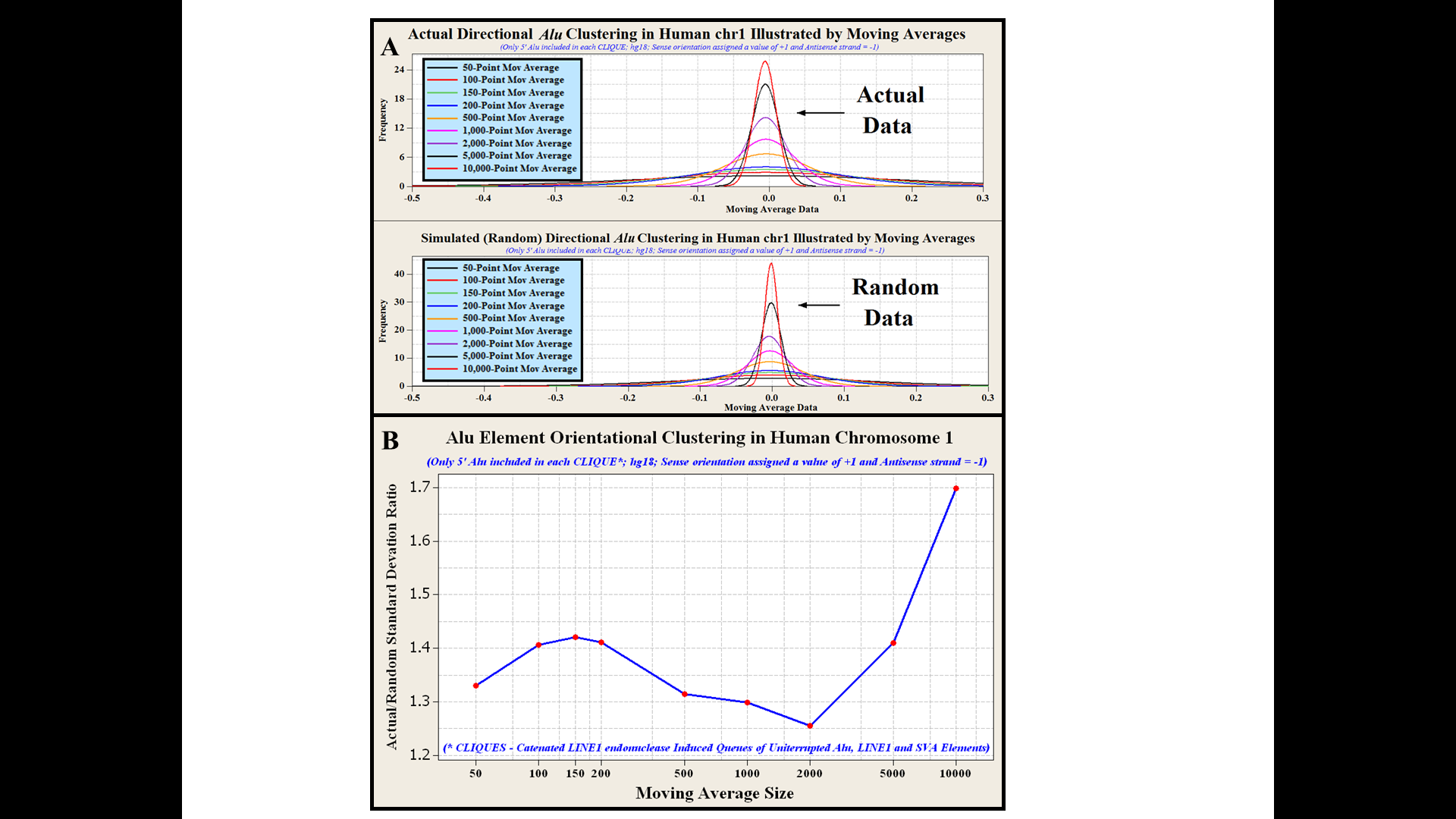
**

**Figure S3.** **Directional *Alu* Element Orientational Clustering in Human Chromosome 1.** Using the RepeatMasker scan of the hg18 human genome assembly, human chromosome 1, chr1, is home to 102,592 *Alu* elements and 34,916 CLIQUEs. *Alu* elements are present in 26,277 of these CLIQUEs. Removing all but the 5’ *Alu* element in CLIQUES (for those CLIQUEs which contain *Alu* elements) reduces the data set used for this chr1 scan to 76,539 *Alus*. *Alu* element orientation was converted to +1 for positive oriented *Alu* elements and -1 for negative oriented elements, and moving averages across chr1 were calculated. A) Distribution of moving average values for actual and random *Alu* clustering data. Note that moving average distributions are less variable for random than for actual data. B) Actual/random standard deviation ratios from the distributions shown in Figure S1-A. Note that except for the extreme cases of moving averages above 2,000, the greatest orientational clustering occurs between APSNs of 100-200. This is consistent with the I:D ratios in Figure 5.

**Figure S4**

**
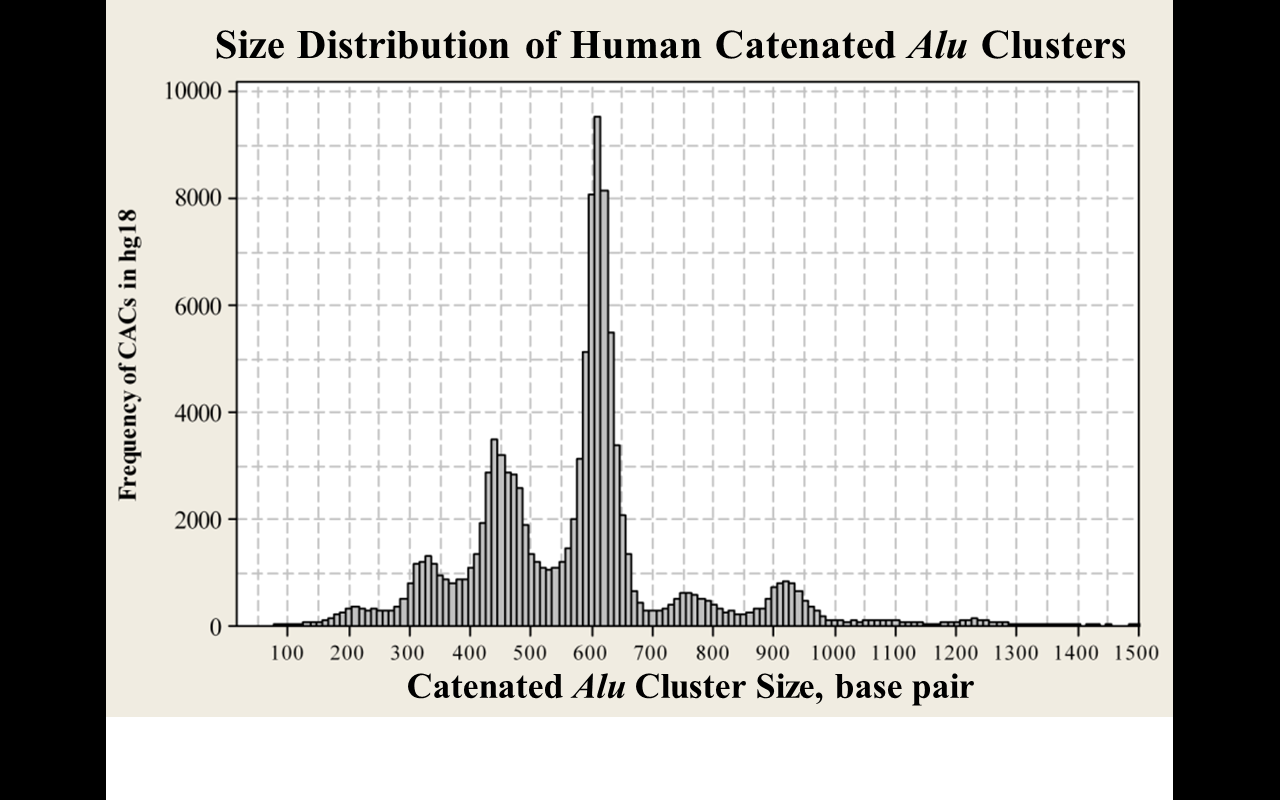
Figure S4. Size distribution for the 113,000 catenated *Alu* clusters (CACs) that reside in the human genome.** CACs are clusters of catenated *Alu* elements which are separated by <50 bp from an adjacent *Alu* element. Approximately 23 percent of all human *Alu* elements and 21 percent of all *Alu* sequence reside in CACs. The average CAC contains 2.4 *Alu* elements, is 567 bp in length and is composed of *Alu* elements having an average length of 238 bp.

**Figure S5**


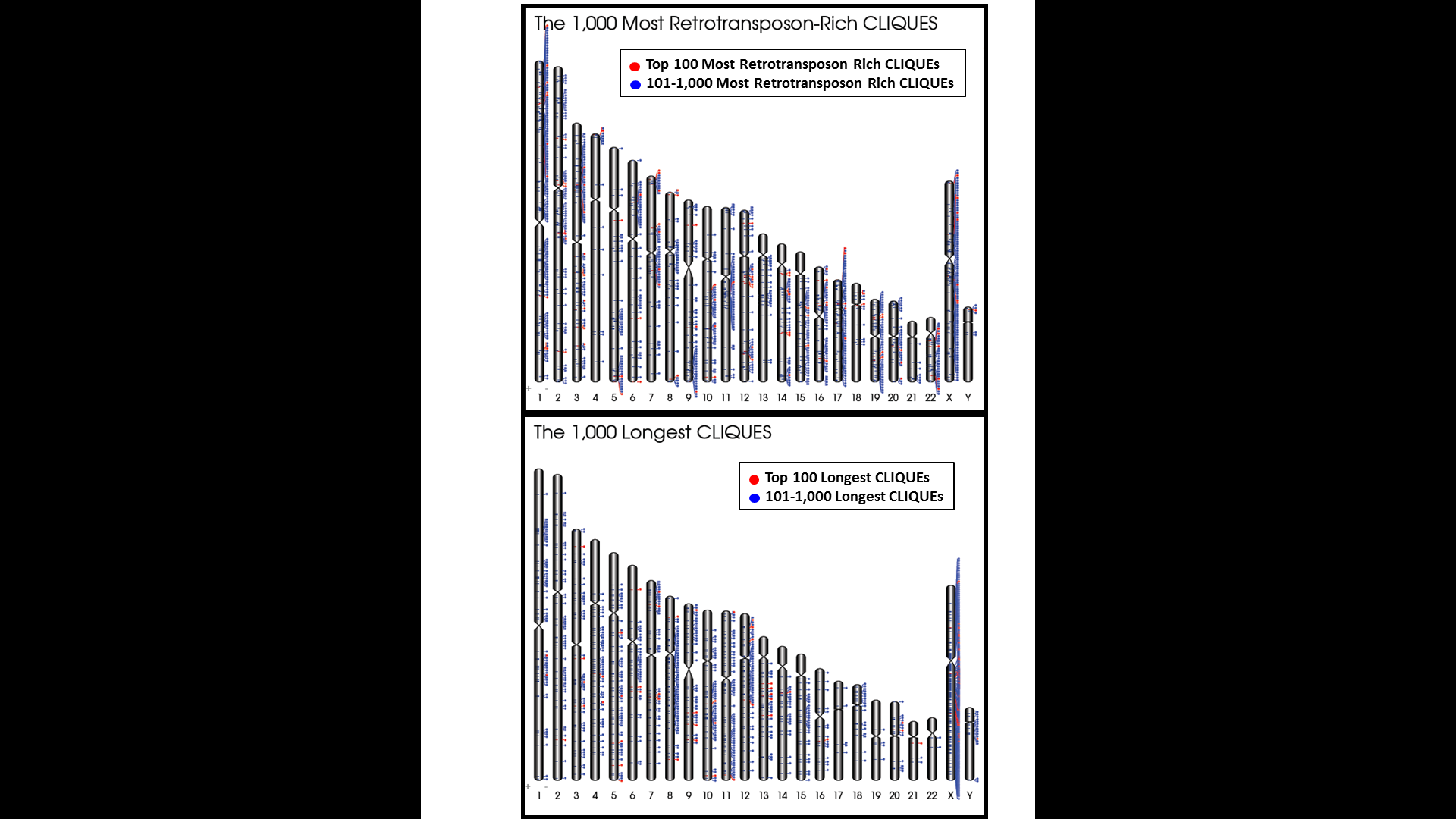
**Figure S5**. **CLIQUE Density Across the Human Genome**. A) The 1,000 most retrotransposon-rich CLIQUEs and B) The 1,000 CLIQUES with the longest sequence. Note that the top 100 most retrotransposon-rich and longest CLIQUEs are denoted in red in each ideogram.

**Figure S6**

**
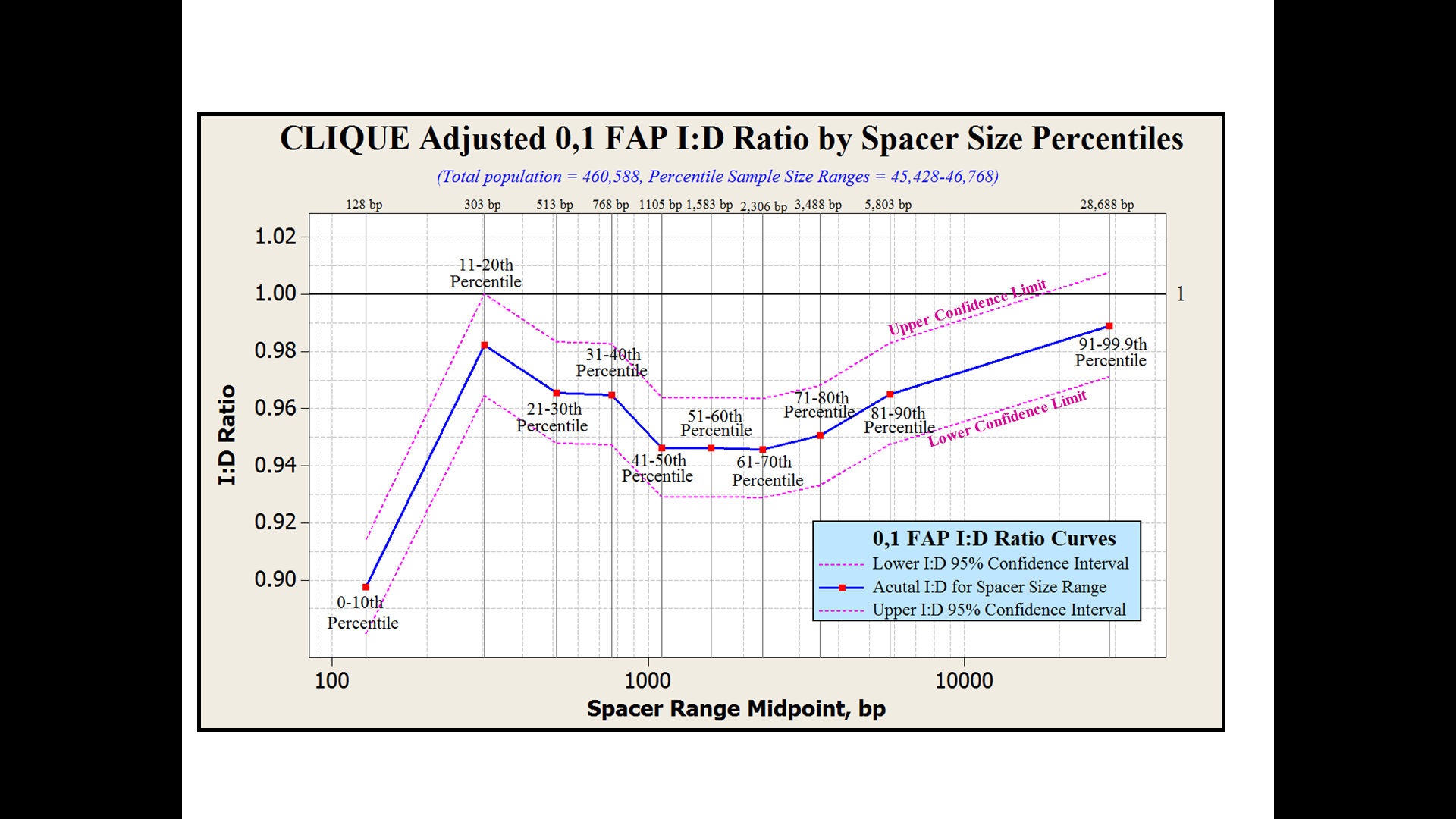
Figure S6. CLIQUE Adjusted 0,1 FAP I:D Ratio by Spacer Size Percentiles.** The CLIQUE adjusted adjacent FAP population is 460,588. This population was broken down into 10 approximately equally-sized groups (size range = 45,428-46,768) based on spacer size. The midpoints of each range are shown along the top border of the graph. The actual I:D ratio for each percentile range is shown (blue) along with the upper and lower boundaries of the 95% confidence interval (red).

**
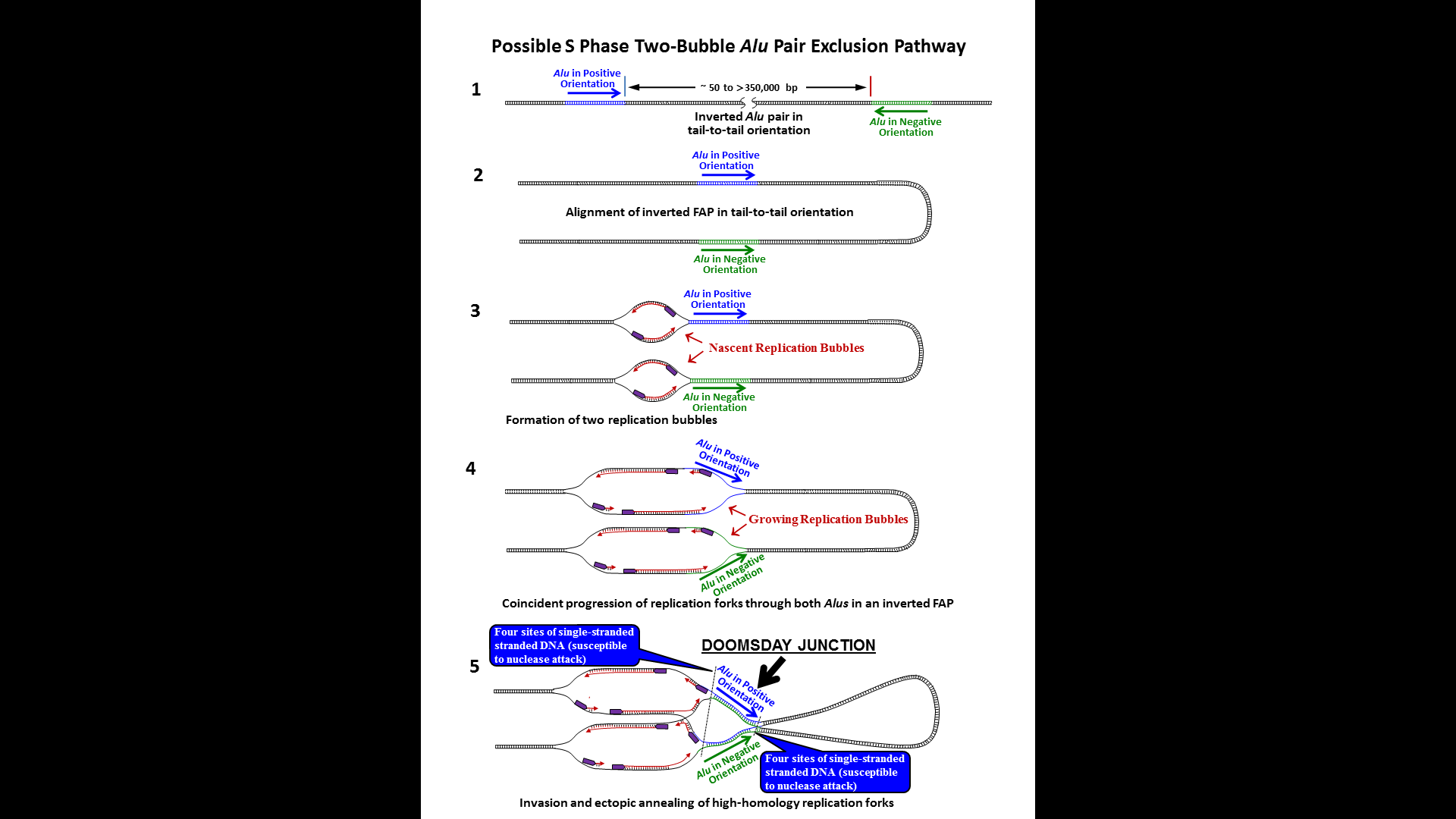
Figure S7**

**Figure S7. Possible S Phase Two-Bubble APE Pathway.** Single-stranded DNA is present at the DNA replication fork during S-phase of the cell cycle. Single-stranded DNA is inherently vulnerable to forming non-canonical binding structures such as hairpins and cruciform structures and thus must be stabilized by single strand binding proteins [[13](#_ENREF_13)]. Figure 6, Steps 1-6B describe the creation of a hypothetical DNA configuration termed a “doomsday junction” or DDJ. The coincident passage and proximity of two separate replication forks through an inverted repeat may set the stage for ectopic invasion and annealing of the single-strand DNA associated with these replication forks. The DDJ pathway described above is similar in all aspects to that outlined in Figure 6 except that the DDJ formation, above, describes a DDJ formation pathway with two DNA replication bubbles.

**Figure S8**


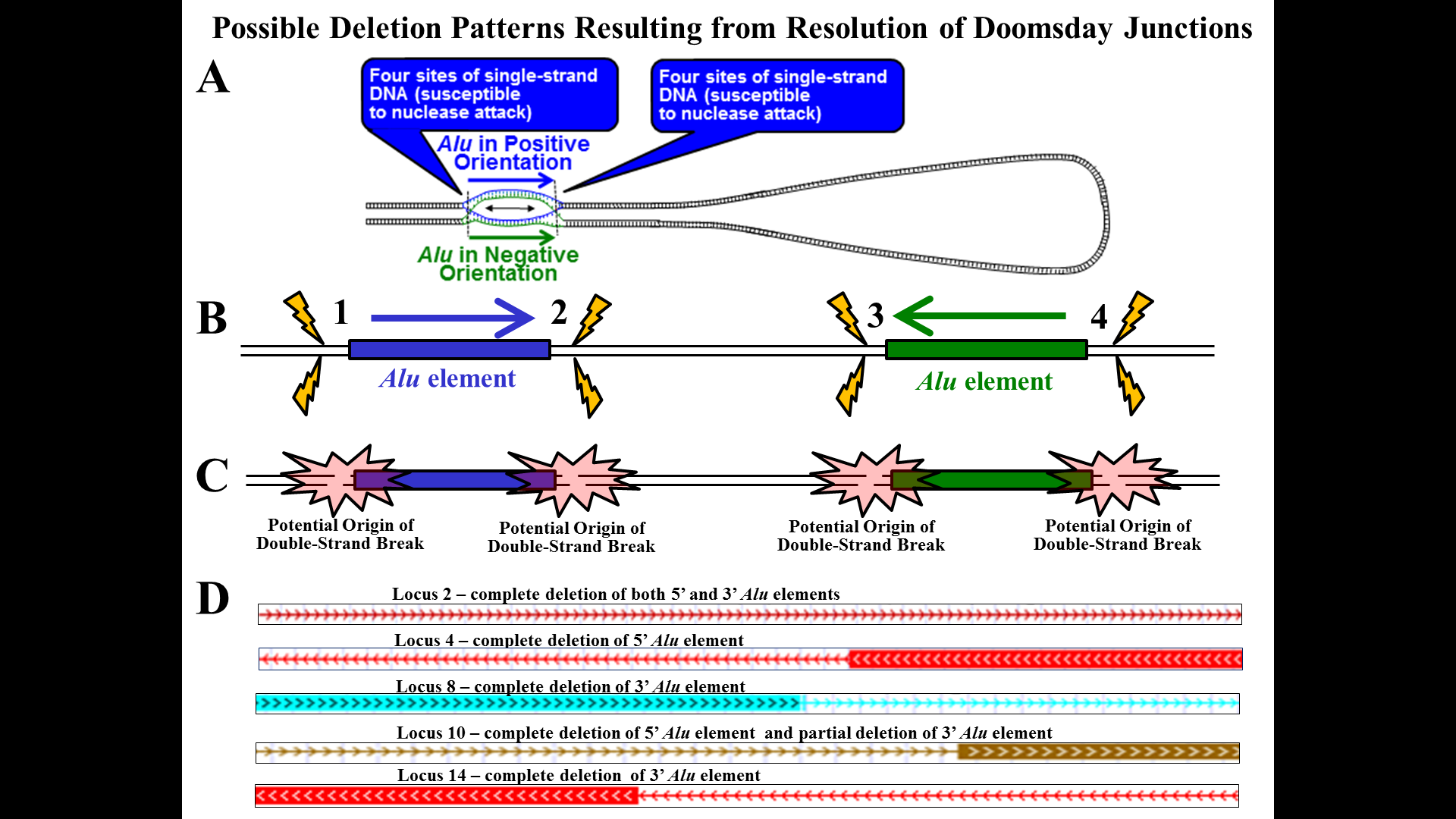


**Figure S8. Possible Deletion Patterns Resulting from Resolution of Doomsday Junctions. A)** The doomsday junction, DDJ, taken from Figure 6, Step 6A. Note the eight regions of single-stranded DNA associated with the ends of the DDJ. These regions may be susceptible to single-strand DNA nuclease attack. B) A linear model of an unraveled DDJ illustrating the eight regions of potential single-strand nuclease attack. C) The regions of the DDJ which are most susceptible to a double-strand break are adjacent to both 5’ and 3’ ends of each *Alu* element (shown as light red starbursts). Using this model, deletion of portions of either *Alu* element or the spacer region would only occur as a result of nuclease attack proceeding from the origin of the double strand break. E) Deletion patterns from PCR chimpanzee loci shown in Figure 6.

**
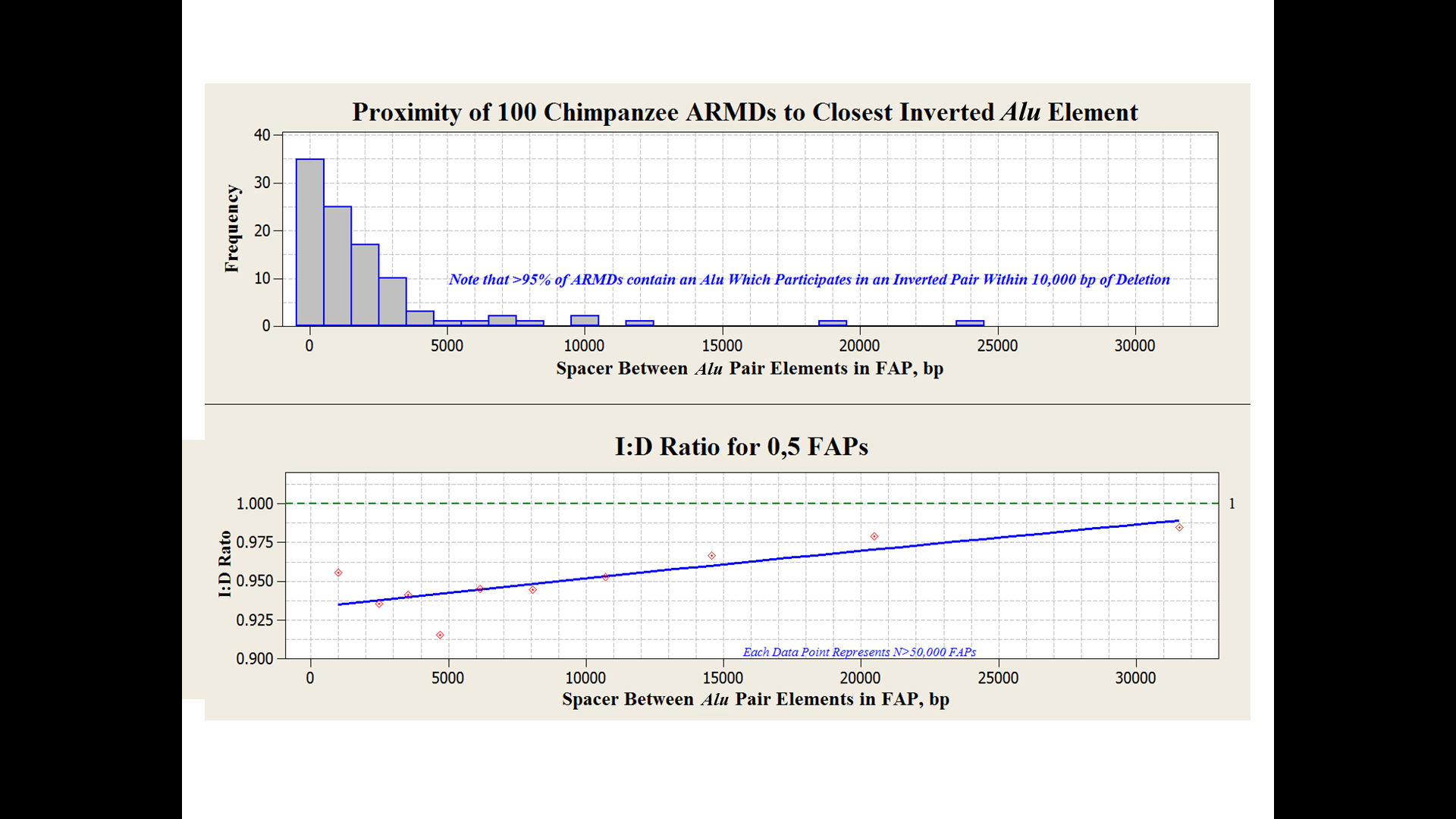
Figure S9**

**Figure S9. ARMDs in Proximity to Inverted *Alu* Pairs.** The cause of indels between chimpanzees and humans can be difficult to diagnose. This is especially true of *Alu* recombination mediated deletions, ARMDs. The existence of the chimeric *Alu* element product of an ARMD provides little information regarding the reasons behind its formation. This chimeric element could be generated by non-allelic homologous recombination, NAHR, or because of homologous repair associated with an unknown deletion. A) The closest inverted *Alu* element for 100 random ARMDs is shown in histogram form. Note that 95% of these ARMDs are within 8,500 bp of an inverted *Alu* element. B) 0,5 FAP I:D ratios were distributed most closely to the scatter seen in these ARMDs. Each data point in this chart represents over 50,000 *Alu* pairs. As can be seen in B) the 95% confidence interval for the I:D ratio about unity is ± 1.7 percent for this sample size. The I:D ratio of 0.95 at a spacer size of 8,500 bp reveals that these ARMDs could be the homologous repair product of a deletion caused by a doomsday junction.

**Table S1**

**Characteristics of CLIQUEs in hg18**

Maximum spacer size between adjacent elements (bp) 50 ^(1)^

Number of CLIQUES in human genome (hg18) 412,380

Fraction of hg18 genome occupied by CLIQUES 16.6 % ^(2)^

Average number of retrotransposons in a CLIQUE 3.3

Average CLIQUE size (base pair) 1,169

Chromosome with Highest CLIQUE Density (fraction of chromosome) ^(3)^ chrX, 28.6%

Chromosome with Lowest CLIQUE Density (fraction of chromosome) ^(3)^ chr21, 15.3%

Chromosome with Highest CLIQUE Density (fraction of total active retrotransposons) ^(4)^ chr19, 68.2%

Chromosome with Lowest CLIQUE Density (fraction of total active retrotransposons) ^(4)^ chr4, 47.3%

*Alu* LINE SVA

Fraction of retrotransposon population within CLIQUEs 49.0 % 55.5% 60.4%

Fraction of retrotransposon sequence within CLIQUEs 47.2% 64.3% 58.5%

Composition of CLIQUEs by sequence 26.8% 72.7% 0.42%

Composition of active retrotransposons in genome by sequence 39.3% 60.3% 0.46%

CLIQUE composition by number of retrotransposons 42.8% 57.1% 0.15%

_____________________________________________________________

1. Defined maximum separation between adjacent *Alu*, LINE and SVA elements within a CLIQUE (see Figure 1).
2. Total CLIQUE sequence divided by the gap-free sequence (2,881,515,245 bp) in the hg18 genome assembly.
3. Total CLIQUE sequence in chromosome divided by the total gap-free sequence of that chromosome (in hg18).
4. Total CLIQUE sequence in chromosome divided by the total *Alu*, LINE and SVA sequence in that chromosome.

**Table S2**

**CLIQUE Adjusted FAP Sample Sizes and I:D, hg18**

| APSN Type | Total  Number | I:D | APSN Type | Total  Number | I:D | APSN Type | Total  Number | I:D | APSN Type | Total  Number | I:D |
| --- | --- | --- | --- | --- | --- | --- | --- | --- | --- | --- | --- |
| 0,1 | 460,588 | 0.9550 | 0,30 | 556,475 | 0.9690 | 0,59 | 556,158 | 0.9830 | 0,88 | 555,764 | 0.9928 |
| 0,2 | 526,986 | 0.9494 | 0,31 | 556,217 | 0.9684 | 0,60 | 556,035 | 0.9887 | 0,89 | 555,471 | 0.9972 |
| 0,3 | 540,117 | 0.9491 | 0,32 | 556,631 | 0.9718 | 0,61 | 556,044 | 0.9897 | 0,90 | 556,080 | 0.9900 |
| 0,4 | 547,346 | 0.9508 | 0,33 | 556,424 | 0.9723 | 0,62 | 556,041 | 0.9899 | 0,91 | 555,560 | 1.0000 |
| 0,5 | 551,764 | 0.9521 | 0,34 | 556,949 | 0.9744 | 0,63 | 556,373 | 0.9884 | 0,92 | 555,753 | 0.9945 |
| 0,6 | 554,173 | 0.9496 | 0,35 | 557,086 | 0.9733 | 0,64 | 556,142 | 0.9869 | 0,93 | 555,742 | 0.9942 |
| 0,7 | 554,928 | 0.9491 | 0,36 | 556,551 | 0.9702 | 0,65 | 556,181 | 0.9865 | 0,94 | 555,439 | 0.9907 |
| 0,8 | 555,811 | 0.9508 | 0,37 | 556,800 | 0.9727 | 0,66 | 555,964 | 0.9929 | 0,95 | 555,643 | 0.9952 |
| 0,9 | 556,349 | 0.9511 | 0,38 | 556,785 | 0.9743 | 0,67 | 556,033 | 0.9876 | 0,96 | 555,501 | 0.9965 |
| 0,10 | 556,963 | 0.9533 | 0,39 | 556,512 | 0.9782 | 0,68 | 555,737 | 0.9837 | 0,97 | 555,354 | 0.9984 |
| 0,11 | 556,857 | 0.9552 | 0,40 | 556,742 | 0.9737 | 0,69 | 555,962 | 0.9848 | 0,98 | 555,539 | 0.9933 |
| 0,12 | 557,454 | 0.9523 | 0,41 | 556,808 | 0.9729 | 0,70 | 555,822 | 0.9843 | 0,99 | 555,980 | 0.9978 |
| 0,13 | 557,033 | 0.9526 | 0,42 | 556,642 | 0.9795 | 0,71 | 555,873 | 0.9859 | 0,100 | 555,392 | 0.9966 |
| 0,14 | 557,023 | 0.9591 | 0,43 | 556,820 | 0.9787 | 0,72 | 556,065 | 0.9877 | 0,101 | 555,340 | 0.9961 |
| 0,15 | 556,948 | 0.9545 | 0,44 | 556,216 | 0.9776 | 0,73 | 555,935 | 0.9942 | 0,102 | 555,491 | 1.0001 |
| 0,16 | 557,239 | 0.9615 | 0,45 | 556,359 | 0.9782 | 0,74 | 555,555 | 0.9945 | 0,103 | 555,697 | 0.9930 |
| 0,17 | 556,970 | 0.9620 | 0,46 | 556,046 | 0.9762 | 0,75 | 555,763 | 0.9900 | 0,104 | 555,014 | 0.9987 |
| 0,18 | 557,002 | 0.9640 | 0,47 | 556,704 | 0.9798 | 0,76 | 556,130 | 0.9938 | 0,105 | 555,082 | 1.0034 |
| 0,19 | 556,886 | 0.9597 | 0,48 | 556,660 | 0.9782 | 0,77 | 556,214 | 0.9926 | 0,106 | 555,165 | 0.9986 |
| 0,20 | 557,127 | 0.9649 | 0,49 | 556,488 | 0.9774 | 0,78 | 555,611 | 0.9857 | 0,107 | 555,588 | 0.9971 |
| 0,21 | 556,925 | 0.9642 | 0,50 | 555,988 | 0.9799 | 0,79 | 555,694 | 0.9912 | 0,108 | 555,104 | 0.9977 |
| 0,22 | 557,364 | 0.9587 | 0,51 | 556,457 | 0.9839 | 0,80 | 555,716 | 0.9957 | 0,109 | 555,298 | 1.0009 |
| 0,23 | 556,997 | 0.9660 | 0,52 | 556,370 | 0.9816 | 0,81 | 555,617 | 0.9946 | 0,110 | 555,168 | 0.9959 |
| 0,24 | 556,822 | 0.9651 | 0,53 | 556,147 | 0.9826 | 0,82 | 555,764 | 0.9945 | 0,111 | 555,536 | 0.9973 |
| 0,25 | 556,542 | 0.9645 | 0,54 | 556,423 | 0.9820 | 0,83 | 555,703 | 0.9891 | 0,112 | 555,117 | 1.0007 |
| 0,26 | 557,104 | 0.9700 | 0,55 | 556,245 | 0.9873 | 0,84 | 555,973 | 0.9895 | 0,113 | 555,699 | 0.9997 |
| 0,27 | 556,690 | 0.9706 | 0,56 | 556,205 | 0.9837 | 0,85 | 555,822 | 0.9918 | 0,114 | 554,985 | 1.0013 |
| 0,28 | 556,952 | 0.9707 | 0,57 | 556,331 | 0.9819 | 0,86 | 555,846 | 0.9915 | 0,115 | 555,514 | 0.9994 |
| 0,29 | 556,469 | 0.9689 | 0,58 | 556,164 | 0.9845 | 0,87 | 555,393 | 0.9898 |  |  |  |

**Table S3**

**Comparison of Orthologous Direct and Inverted FAP Loci**^(1)^

**Direct FAP Loci Inverted FAP Loci**

**Loci Characteristics (Number, %) (Number, %)**

**Orthologous panTro2/hg18 FAP Loci** ^(2)^

Total orthologous FAP loci 14,680, 100% 13,664, 100%

PanTro2 loci 1,000-2,000 bp shorter than hg18 orthologue 193, 1.2% 186, 1.4%

**Examination of Shorter Chimp Loci**

**1 – Human-Specific Retrotransposon or Repetitive DNA Insertion(s)**  72, 37.3% 45, 24.2%

**2 - Possible APE-Related Deletions**

A-Possible interaction of inverted *Alu* pair associated with indel ^(3)^ 95, 49.2% 106, 57.0%

B-Inverted L1EN consensus Target Site(s) within human/chimp indel ^(4)^ 22, 11.4% 31, 16.7%

**3 – Possible non-*Alu* Inverted Sequence Deletions**

C-Palindrome (with spacer) within human/chimp indel ^(5)^ 4, 2.1% 4, 2.2%

**Potential APEs Resulting in *Alu-Alu* SSA ^(6)^ Repair, (% of APEs)** 15^(7)^ (16.1%) 5^(7)^ (4.7%)

**__________ _________________________**

1. panTro2 loci which are 1,000-2,000 bp shorter than the orthologous loci in hg18.
2. Orthologous loci have hg18 spacer sizes between 651-1,500 bp and 1,000 bp of 5’ and 3’ “*Alu* element free,” flanking sequence.
3. Approximately half of the shorter chimpanzee direct FAP loci had deletion patterns that were consistent with inverted APE deletions (Figure S8). These potential APE deletions could result from the instability of a second inverted *Alu* pair formed by a flanking *Alu* element and one of the *Alu* elements within the FAP being evaluated.
4. One or more L1EN target site sequences (5’-TTTTAA-3’) is/are present in the orthologous human sequence of the chimpanzee deletion. These orthologous target sites are in the inverted orientation relative to an existing *Alu* present within the loci window. The presence of L1EN inverted target site(s) within this human/chimpanzee orthologous indel opens the possibility that the indel may be the result of a chimpanzee-specific APE deletion catalyzed by a chimpanzee-specific *Alu* insertion.
5. A palindrome of minimum length of 7 bp was present in the orthologous human sequence of the chimpanzee deletion. This palindrome could create a potential region of instability within the deletion. This instability could possibly occur by a mechanism similar to those outlined in Figures 6 and S7.
6. SSA – Single Strand Annealing repair (Hedges et al., 2007).
7. The incorporation of a direct-oriented *Alu* pair into the SSA repair of a deletion event can produce a chimeric *Alu* element (Sen et al., 2006). The examination of these direct and inverted FAP loci revealed that several chimeric *Alu* elements apparently resulted from these potential chimpanzee APE-related deletions. The number of chimeric *Alu* elements produced from these events is shown here along with the percentage as a total of potential APE-related deletions (see Supplemental Information, Potential for ARMD Masking of APE Deletions and Figure S9).

**Table S4**

**Primers for Selected APE Loci in Listed in Table 1**

**(Orthologous in Human, Chimpanzee, Gorilla, Orangutan and Rhesus macaque)**

**Inverted Spacer**

**Loci Forward Primer Reverse Primer Temperatures *Alu* FAP Size**

**ID hg18 Position ( 5’ to 3’) (5’ to 3’) Annealing Extending Pair Orientation^(1)^ (bp)**

1 chr1:105842254-105848252 GGAAAGTGGATATCCTTTGGG TTGTTCATTGTTCCTTTTAATT 50°C 68°C *AluY- AluJb* Tail-to-Tail 1,407

2 chr4:54368003-54376671 CCTCATGTCCTCCCCTTTAC CACCATGAGCTCATCCTATGC 50°C 68°C *AluSx- AluSx* Head-to-Head 1,292

3 chr2:68246922-68253405 CATCGAGTTCTCTTCCATAGC CCTGAAAAGGGTGAAATGGAG 50°C 68°C *AluY- AluY* Head-to-Head 1,237

4 chr5:71966234-71974703 GGCAAATCCTGTTTCACCACC GGAAACGAGGCTAAATAATGGC 62°C 68°C *AluSq-AluSg* Head-to-Head 1,012

5 chr13:64130795-64137788 CTACATAAGCTTGCACTTCTTTG AGTAAGAAAGCTGGTTCTGAAGA 50°C 68°C *AluJo- AluSx* Tail-to-Tail 1,312

8 chr17:65716901-65723822 GGGAAAATTGTTTCTGTACAGGG CACATGCTGAGAAGCCACTAC 50°C 68°C *AluSg- AluY* Tail-to-Tail 1,285

9 chr8:53032075-53037664 GTCAGTCCACCAAGGTGGTTA CCCTTAAAACATATCTGGAATCATC 50°C 68°C *AluSx- AluSx* Tail-to-Tail 973

10 chr1:16,314,268-16,319,666 GATCTGGCCCTAGATTTGACAG GCCTGTTCCTAGAGGAGTTGC 62°C 68°C *AluSg- AluSq* Tail-to-Tail 793

14 chr5:78401563-78406842 GGTAGTTAGAATAGCAGTGAAGG GCAGAAAGGAGTTTAATATTGAG 55°C 68°C *AluSq- AluSx* Tail-to-Tail 665

15 chr4:68494452-68500177 GGAATGGTTTCTCTTAGCAGC GTGAGATCCTGAGCAGAAAGC 60°C 68°C *AluY- AluSx* Head-to-Head 1,121

_________________________________________________________________

1. When an inverted *Alu* pair is oriented with the poly(A) tails pointing toward each other, the pair is defined as being in the “Tail-to-Tail” orientation, and when an inverted pair is oriented with the poly(A) tails pointing away from each other, it is defined as being in the “Head-to-Head” orientation.

**References**

1. Levy A, Schwartz S, Ast G: **Large-scale discovery of insertion hotspots and preferential integration sites of human transposed elements.** *Nucleic Acids Res* 2010, **38:**1515-1530.

2. Lobachev KS, Stenger JE, Kozyreva OG, Jurka J, Gordenin DA, Resnick MA: **Inverted Alu repeats unstable in yeast are excluded from the human genome.** *EMBO J* 2000, **19:**3822-3830.

3. Srikanta D, Sen SK, Conlin EM, Batzer MA: **Internal priming: an opportunistic pathway for L1 and Alu retrotransposition in hominins.** *Gene* 2009, **448:**233-241.

4. Cordaux R, Batzer MA: **The impact of retrotransposons on human genome evolution.** *Nat Rev Genet* 2009, **10:**691-703.

5. Shaikh TH, Roy AM, Kim J, Batzer MA, Deininger PL: **cDNAs derived from primary and small cytoplasmic Alu (scAlu) transcripts.** *J Mol Biol* 1997, **271:**222-234.

6. Orioli A, Pascali C, Quartararo J, Diebel KW, Praz V, Romascano D, Percudani R, van Dyk LF, Hernandez N, Teichmann M, Dieci G: **Widespread occurrence of non-canonical transcription termination by human RNA polymerase III.** *Nucleic Acids Res* 2011.

7. Lander ES, Linton LM, Birren B, Nusbaum C, Zody MC, Baldwin J, Devon K, Dewar K, Doyle M, FitzHugh W, et al: **Initial sequencing and analysis of the human genome.** *Nature* 2001, **409:**860-921.

8. Weichenrieder O, Repanas K, Perrakis A: **Crystal structure of the targeting endonuclease of the human LINE-1 retrotransposon.** *Structure* 2004, **12:**975-986.

9. Stenger JE, Lobachev KS, Gordenin D, Darden TA, Jurka J, Resnick MA: **Biased distribution of inverted and direct Alus in the human genome: implications for insertion, exclusion, and genome stability.** *Genome Res* 2001, **11:**12-27.

10. Hedges DJ, Deininger PL: **Inviting instability: Transposable elements, double-strand breaks, and the maintenance of genome integrity.** *Mutat Res* 2007, **616:**46-59.

11. Sen SK, Han K, Wang J, Lee J, Wang H, Callinan PA, Dyer M, Cordaux R, Liang P, Batzer MA: **Human genomic deletions mediated by recombination between Alu elements.** *Am J Hum Genet* 2006, **79:**41-53.

12. Han K, Lee J, Meyer TJ, Wang J, Sen SK, Srikanta D, Liang P, Batzer MA: **Alu recombination-mediated structural deletions in the chimpanzee genome.** *PLoS Genet* 2007, **3:**1939-1949.

13. Broderick S, Rehmet K, Concannon C, Nasheuer HP: **Eukaryotic single-stranded DNA binding proteins: central factors in genome stability.** *Subcell Biochem* 2010, **50:**143-163.
