# Supplementary figures and images for: Alu pair exclusions in the human genome
Source: Mob DNA. 2011 Sep 23;2:10. doi: 10.1186/1759-8753-2-10 (PMC3215922; doi:10.1186/1759-8753-2-10)

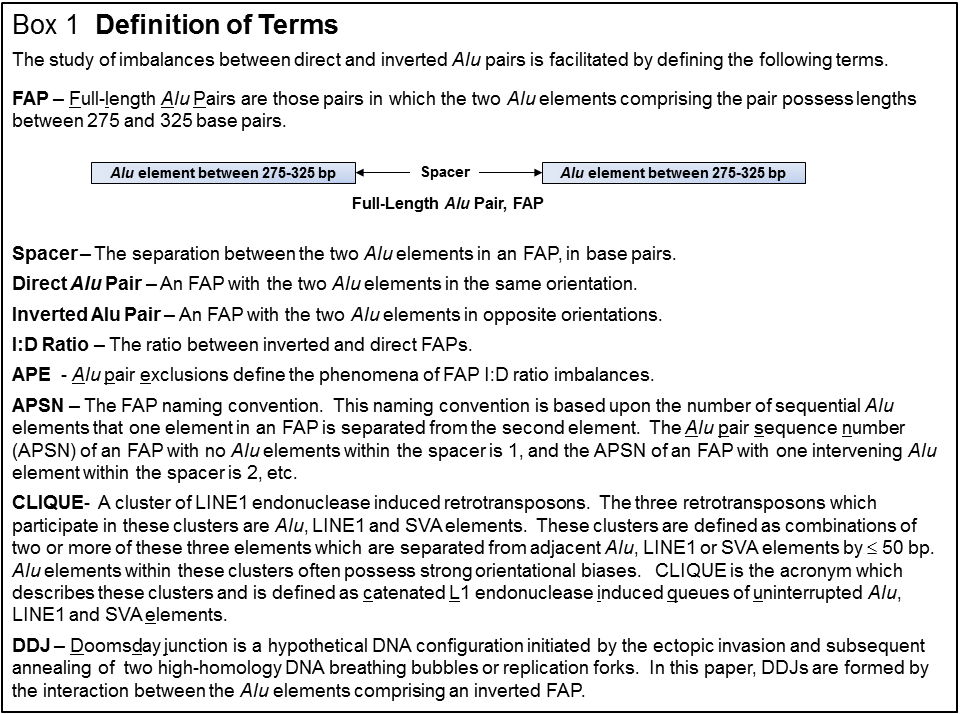

Supplement: Additional file 2 — Definition of Terms. This file contains a list with definitions of abbreviations and novel terminology introduced within the manuscript. [file 1759-8753-2-10-S2.TIFF]
